# Functional divergence of chloroplast Cpn60α subunits during *Arabidopsis* embryo development

**DOI:** 10.1371/journal.pgen.1007036

**Published:** 2017-09-29

**Authors:** Xiaolong Ke, Wenxuan Zou, Yafang Ren, Zhiqin Wang, Jin Li, Xuan Wu, Jie Zhao

**Affiliations:** State Key Laboratory of Hybrid Rice, College of Life Sciences, Wuhan University, Wuhan, China; John Innes Centre, UNITED KINGDOM

## Abstract

Chaperonins are a class of molecular chaperones that assist in the folding and assembly of a wide range of substrates. In plants, chloroplast chaperonins are composed of two different types of subunits, Cpn60α and Cpn60β, and duplication of *Cpn60α* and *Cpn60β* genes occurs in a high proportion of plants. However, the importance of multiple *Cpn60α* and *Cpn60β* genes in plants is poorly understood. In this study, we found that loss-of-function of *CPNA2* (*AtCpn60α2*), a gene encoding the minor Cpn60α subunit in *Arabidopsis thaliana*, resulted in arrested embryo development at the globular stage, whereas the other *AtCpn60α* gene encoding the dominant Cpn60α subunit, *CPNA1* (*AtCpn60α1*), mainly affected embryonic cotyledon development at the torpedo stage and thereafter. Further studies demonstrated that CPNA2 can form a functional chaperonin with CPNB2 (AtCpn60β2) and CPNB3 (AtCpn60β3), while the functional partners of CPNA1 are CPNB1 (AtCpn60β1) and CPNB2. We also revealed that the functional chaperonin containing CPNA2 could assist the folding of a specific substrate, KASI (β-ketoacyl-[acyl carrier protein] synthase I), and that the KASI protein level was remarkably reduced due to loss-of-function of *CPNA2*. Furthermore, the reduction in the KASI protein level was shown to be the possible cause for the arrest of *cpna2* embryos. Our findings indicate that the two Cpn60α subunits in *Arabidopsis* play different roles during embryo development through forming distinct chaperonins with specific AtCpn60β to assist the folding of particular substrates, thus providing novel insights into functional divergence of Cpn60α subunits in plants.

## Author summary

Chaperonins are large oligomeric complexes that are involved in the folding and assembly of numerous proteins in various species. In contrast to other types of chaperonins, chloroplast chaperonins are characterized by the hetero-oligomeric structure composed of two unique types of subunits, Cpn60α and Cpn60β, each of which is present in two or more paralogous forms in most of higher plants. However, the functional significance underlying the wide array of subunit types and complex oligomeric arrangement remains largely unknown. Here, we investigated the role of the minor Cpn60α subunit AtCpn60α2 in *Arabidopsis* embryo development, and found that AtCpn60α2 is important for the transition of globular embryos to heart-shaped embryos, whereas loss of the dominant Cpn60α subunit AtCpn60α1 affects embryonic cotyledon development. Further studies demonstrated that AtCpn60α2 could form functional chaperonins with AtCpn60β2 and AtCpn60β3 to specifically assist in folding of the substrate KASI, which is important for the formation of heart-shaped embryos. Our results suggest that duplication of *Cpn60α* genes in higher plants can increase the potential number of chloroplast chaperonin substrates and provide chloroplast chaperonins with more roles in plant growth and development, thus revealing the relationship between duplication and functional specialization of chaperonin genes.

## Introduction

Chaperonins are a class of molecular chaperones that are characterized by a barrel-shaped architecture formed by two stacked oligomeric rings consisting of several subunits of approximately 60 kDa. Two types of chaperonins have been identified: type I chaperonins are found in eubacteria, chloroplasts, and mitochondria; and type II exist in archaea and eukaryotic cytosol. The main difference between them is that type I chaperonins require co-chaperonins consisting of seven 10 kDa subunits for substrate encapsulation, whereas type II chaperonins have a built-in lid that plays the same role [1]. The GroEL/GroES complex in *Escherichia coil* has been studied extensively as the prototype of type I chaperonins. GroEL is a homo-oligomer which consists of two stacked heptameric rings. The folding cycle in GroEL/GroES has been surveyed in detail, and the canonical view suggested that the complex operates through the asymmetric “bullet” cycle. In this asymmetric cycle, unfolded/misfolded substrates first bind to the hydrophobic cavity lining of one ring (*cis* ring), utilizing the exposed hydrophobic residues, and then the binding of ATP causes large conformational changes of the *cis* ring that further trigger the binding of co-chaperonins. Binding of co-chaperonins initiates further conformational changes and caps the *cis* ring, and consequently encapsulates the substrates into an expanded cavity with a hydrophilic lining, which assists the substrates to refold into their native states. Following the hydrolysis of ATP in the *cis* ring, ATP and other non-native proteins bind to the opposite ring (*trans* ring), resulting in the dissociation of refolded substrates, ADP, and co-chaperonins in the *cis* ring [2–4]. Moreover, an alternative model, known as the symmetric “football” model, was recently also proposed. In this model, the exchange of ADP to ATP is extremely rapid in the presence of abundant substrate protein, resulting in formation of a symmetric “football” intermediate that has GroES bound to both rings and can assist in protein folding simultaneously in both rings. This “football” intermediate would be reverted to the “bullet” conformation upon ATP hydrolysis [5–7]. In addition, although *Escherichia coil* only contains one chaperonin gene, a survey of 669 complete bacterial genomes showed that nearly 30% contain two or more chaperonin genes, and a degree of subfunctionalization has occurred in the chaperonin subunits encoded by these duplicated genes [8]. Moreover, in the previous study, Wang and coworkers found that some specific mutations of GroEL can improve the folding of GFP, but the mutated GroEL has a reduced ability to function as general chaperones, suggesting a conflict between the increased ability of GroEL to fold particular substrates and its general ability to fold a wide range of substrates, and this conflict would be resolved by duplication and variation of chaperonin genes [9].

Chloroplast chaperonin (Ch-Cpn60) was first found as a homolog of GroEL that could bind to the chloroplast Rubisco large subunit and assist the assembly of Rubisco, a key rate-limiting enzyme in the process of carbon dioxide fixation [10]. In contrast to GroEL, ch-Cpn60s contain two different types of subunits, Cpn60α and Cpn60β, which only share approximately 50% identity. Ch-Cpn60s composed of Cpn60α and Cpn60β are considered to be the native form of chloroplast chaperonins *in vivo*, because ch-Cpn60s purified from *Pisum sativum*, *Brassica napus*, *Arabidopsis thaliana* and *Spinacia oleracea* were all shown to be hetero-oligomers consisting of nearly equal amounts of Cpn60α and Cpn60β [11–13]. In *Arabidopsis thaliana*, there are two *Cpn60α* genes and four *Cpn60β* genes, which encode three dominant subunits: AtCpn60α1, AtCpn60β1 and AtCpn60β2; and three minor subunits: AtCpn60α2, AtCpn60β3 and AtCpn60β4 (the nomenclature used in this article is in accordance with The *Arabidopsis* Information Resource database). Among them, AtCpn60α1 and AtCpn60α2 share only about 57% identity, and AtCpn60β1/2/3 share 90%-95% identity, while AtCpn60β4 is only 60% identical to the other AtCpn60β subunits [14–15]. *AtCpn60α1* was the first chaperonin gene studied in detail, and its mutant, *schlepperless* (*slp*), showed retardation of embryo development before the heart stage, and defective embryos with highly reduced cotyledons [16]. Then a T-DNA insertion mutant of *AtCpn60α2*, *emb3007*, showed the embryo development arrested at the globular stage in the SeedGenes database (http://www.seedgenes.org/), suggesting that *AtCpn60α2* is also possibly an embryo-defective gene [17–18]. A T-DNA mutant lacking the *AtCpn60β1* transcript, *len1*, had impaired leaves and showed systemic acquired resistance (SAR) under short-day condition [19]. It was also reported that a weak mutant of *AtCpn60α1* and a strong mutant allele of *AtCpn60β1* both showed impaired chloroplast division and reduced chlorophyll levels, and the *AtCpn60β1 AtCpn60β2* double mutant led to an albino seedling similar to *slp*, suggesting that AtCpn60β1 and AtCpn60β2 are redundantly required for normal chloroplast function, together with AtCpn60α1 [20]. In addition, a recent report also showed that the ch-Cpn60 containing the AtCpn60β4 subunit played a specific role in the folding of NdhH, a subunit of the chloroplast NADH dehydrogenase-like complex (NDH), indicating that the particular type of AtCpn60β subunit could contribute to the folding of some specific substrates [21].

Embryogenesis is the beginning of plant development. During *Arabidopsis* embryo development, chloroplast biogenesis is a temporary process. Proplastids in the whole embryo first begin to differentiate into chloroplasts at the transition stage, and then the mature chloroplasts degenerate to undifferentiated eoplasts during seed maturation [22–24]. For decades, through forward and reverse genetic screens in *Arabidopsis*, numerous chloroplast proteins crucial for embryo development were discovered. Interestingly, nearly all embryo defects caused by chloroplast dysfunction displayed premature arrest at the globular stage, indicating that the formation of impermanent chloroplasts in *Arabidopsis* embryos is especially crucial for the transition of globular embryos to heart-shaped embryos [25–27].

Here, we provided genetic evidence to show that the two *AtCpn60α* genes in *Arabidopsis* affect the embryonic development at different stages. Further studies revealed that CPNA2 could form a functional chaperonin with AtCpn60β2 and AtCpn60β3 subunits to specifically assist the folding of KASI (β-ketoacyl-[acyl carrier protein] synthase I), and KASI could not be folded by the functional chaperonin containing CPNA1. Moreover, we found that the KASI protein level was largely reduced due to loss-of-function of *CPNA2*, and the reduction in KASI protein level possibly caused the abnormality of the *cpna2* embryos. Our results showed that Cpn60α2 and Cpn60α1 in *Arabidopsis* can form functional chaperonin complexes with specific AtCpn60β subunits to assist the folding of particular substrates, indicating that functional divergence of chloroplast Cpn60α subunits has occurred in higher plants.

## Results

### Identification of an embryo-defective (*EMB*) gene *CPNA2* in *Arabidopsis*

**Fig 1:**
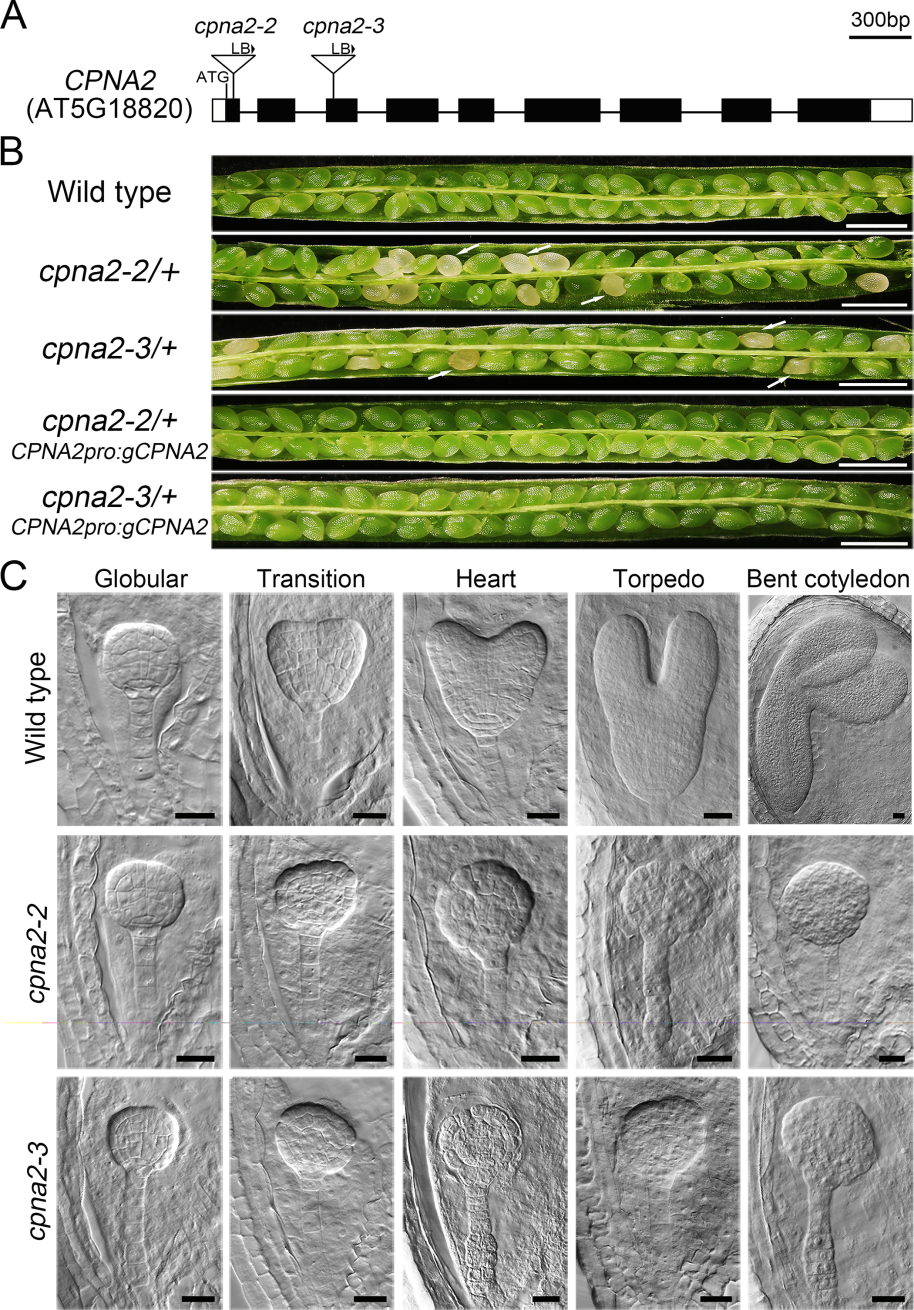
Characterization of *cpna2* mutants. (A) Schematic diagram of the *CPNA2* gene structure with the positions of T-DNA insertion. Exons are shown as black boxes, untranslated regions as white boxes, introns and the promoter as lines; LB indicates left border primer of T-DNA. (B) Silique phenotypes of *cpna2* mutants and complemented mutants. Arrows indicate abnormal ovules. Bars = 1 mm. (C) Phenotypic observation of embryos from wild-type plants and embryos in abnormal ovules from *cpna2-2*/+ and *cpna2-3*/+ plants. Bars = 20 μm.

To elucidate the molecular mechanisms that control embryo development in *Arabidopsis*, we ordered the stock CS76507, which is a set of 10,000 T-DNA lines, from *Arabidopsis* Biological Resource Center (ABRC, http://abrc.osu.edu/). From the stock, we obtained a mutant that showed obvious seed abortion, with a frequency of 25.55% (n = 2270). We found that the T-DNA insertion of this mutant is located in the first exon of AT5G18820 (Fig 1A) using thermal asymmetric interlaced PCR [28]. We named the gene *CPNA2* because it encodes the chaperonin subunit AtCpn60α2, and named the mutant *cpna2-2* (The *emb3007* mutant described previously was designated as *cpna2-1* here). PCR analysis of *cpna2-2*/+ progeny showed that no homozygous mutant plant existed, and the ratio of heterozygote to wild type was nearly 2:1 (S1 Table). Reciprocal crosses between heterozygote and wild-type plants further demonstrated that the transmission efficiency of gametophytes was not affected by loss-of-function of *CPNA2* (S1 Table). Moreover, we obtained another T-DNA mutant (*cpna2-3*, SALK_144574) from ABRC, and found that the mutant *cpna2-3*/+ also showed seed abortion, with a frequency of 26.09% (n = 1196) (Fig 1A and 1B).

To further confirm that *CPNA2* is responsible for seed abortion in *cpna2-2*/+ and *cpna2-3*/*+* plants, we performed a complementation test. A genomic fragment, including *CPNA2*, 1548 bp upstream of the start codon and 673 bp downstream of *CPNA2*, was introduced into *cpna2-2*/+ and *cpna2-3*/+. The result showed that the fertility was restored in the siliques of *CPNA2pro*:*gCPNA2* transgenic plants (Fig 1B). Together, these results indicated that seed abortion in *Arabidopsis* could be caused by loss-of-function of *CPNA2*.

**Fig 2:**
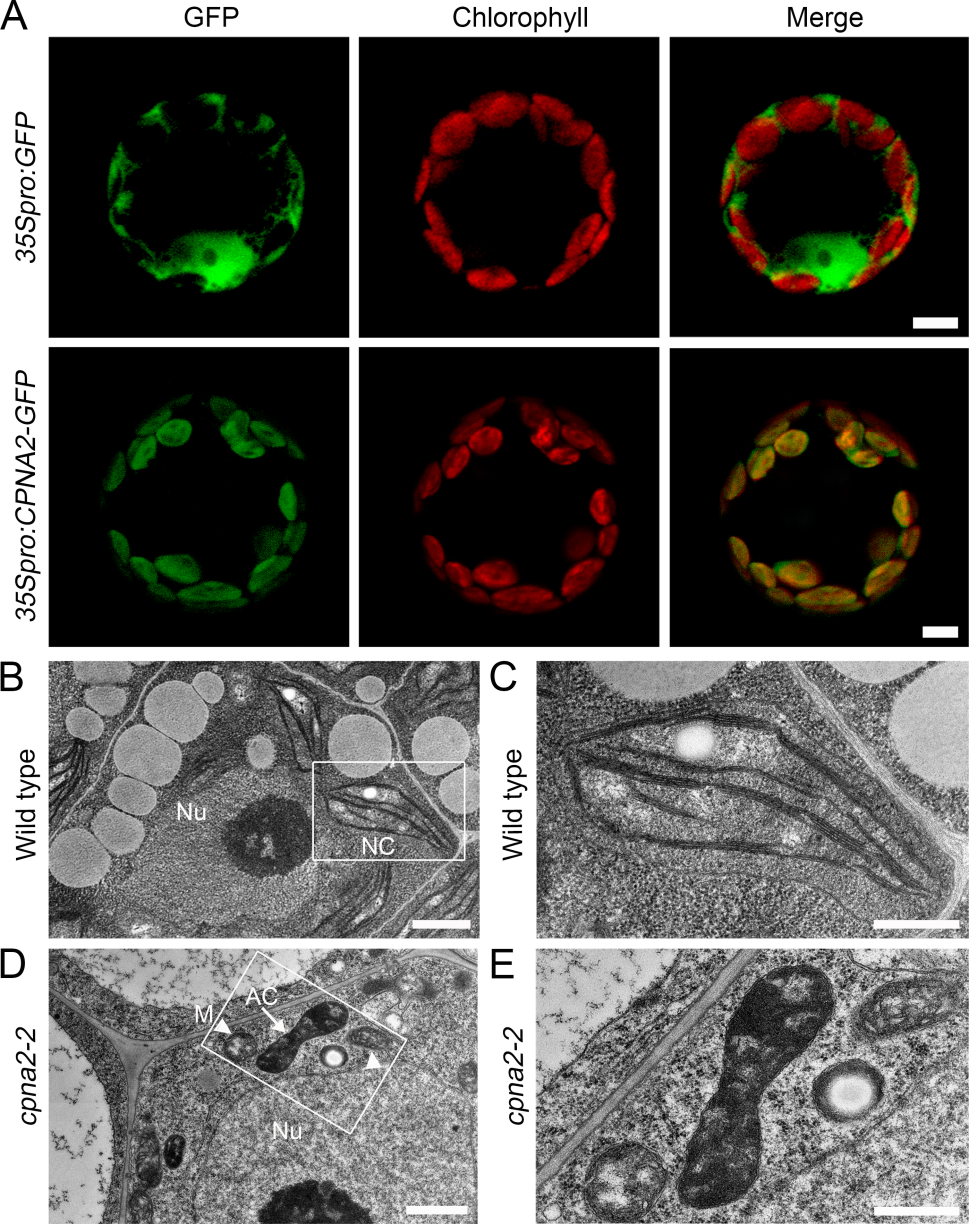
Subcellular localization of CPNA2 and ultrastructure of chloroplasts in wild-type and *cpna2-2* embryos. (A) Fluorescent signals in mesophyll protoplasts of *35Spro*:*GFP* and *35Spro*:*CPNA2-GFP* transgenic plants. Bars = 5 μm. (B-E) Transmission electron microscopy of chloroplasts in 6 DAP wild-type (B and C) and *cpna2-2* (D and E) embryos. (C) and (E) are the magnification of white frames in (B) and (D), respectively. The arrow indicates the abnormal chloroplast; arrowheads indicate mitochondria; Nu, nucleus; NC, normal chloroplast; M, mitochondria; AC, abnormal chloroplast. Bars in (B) and (D) = 1 μm, Bars in (C) and (E) = 0.5 μm.

To investigate the cause of seed abortion of *cpna2-2*/+ and *cpna2-3*/+, we first examined the processes of embryo development in the seeds of *cpna2-2*/+ and *cpna2-3*/+ using the whole mount clearing technique. We could not distinguish abnormal embryos in the siliques from the zygote stage to the globular stage. However, when most embryos in the seeds of *cpna2-2*/+ and *cpna2-3*/+ reached the transition stage, some embryos showed the irregular globular shape and the start of abnormal cell division (Fig 1C). While wild-type embryos progressed into further stages, the abnormal embryos still stayed at the globular stage, and finally degraded along with the collapse of seeds (Fig 1C). The phenotype of these abnormal embryos is consistent with the mutant *emb3007* in SeedGenes (http://www.seedgenes.org/; [17–18]). In addition, since *CPNA2* was predicted to encode a chloroplast chaperonin subunit and chloroplast chaperonins had been reported to be involved in the folding and assembly of many chloroplast proteins [29], we wondered whether the abortion of *cpna2* embryos was due to impaired chloroplast development. To investigate this, we first examined the subcellular localization of CPNA2 in mesophyll protoplasts isolated from transgenic plants carrying the *35Spro*:*CPNA2-GFP* construct. As shown in Fig 2A, the protoplasts containing CPNA2-GFP fusion protein displayed GFP signals that overlapped with chlorophyll autofluorescence, confirming that CPNA2 is located in chloroplasts. Then we observed the ultrastructure of chloroplasts using transmission electron microscopy. In the 6 DAP siliques of *cpna2-2*/+ plant, we observed that mature chloroplasts in wild-type embryos contained organized thylakoid membranes stacked into grana (Fig 2B and 2C). In contrast, only abnormal chloroplasts that lacked thylakoid membranes and contained a deeply stained mass were found in *cpna2-2* mutant embryos (Fig 2D and 2E). Collectively, these results suggested that loss-of-function of *CPNA2* impeded the process by which proplastids differentiate into mature chloroplasts during embryo development, thereby causing the arrest of the *cpna2* embryos and seed abortion of *cpna2-2*/+ and *cpna2-3*/+.

### *CPNA2* is highly expressed in the shoot apical meristem (SAM) of early seedlings and embryonic cotyledons

**Fig 3:**
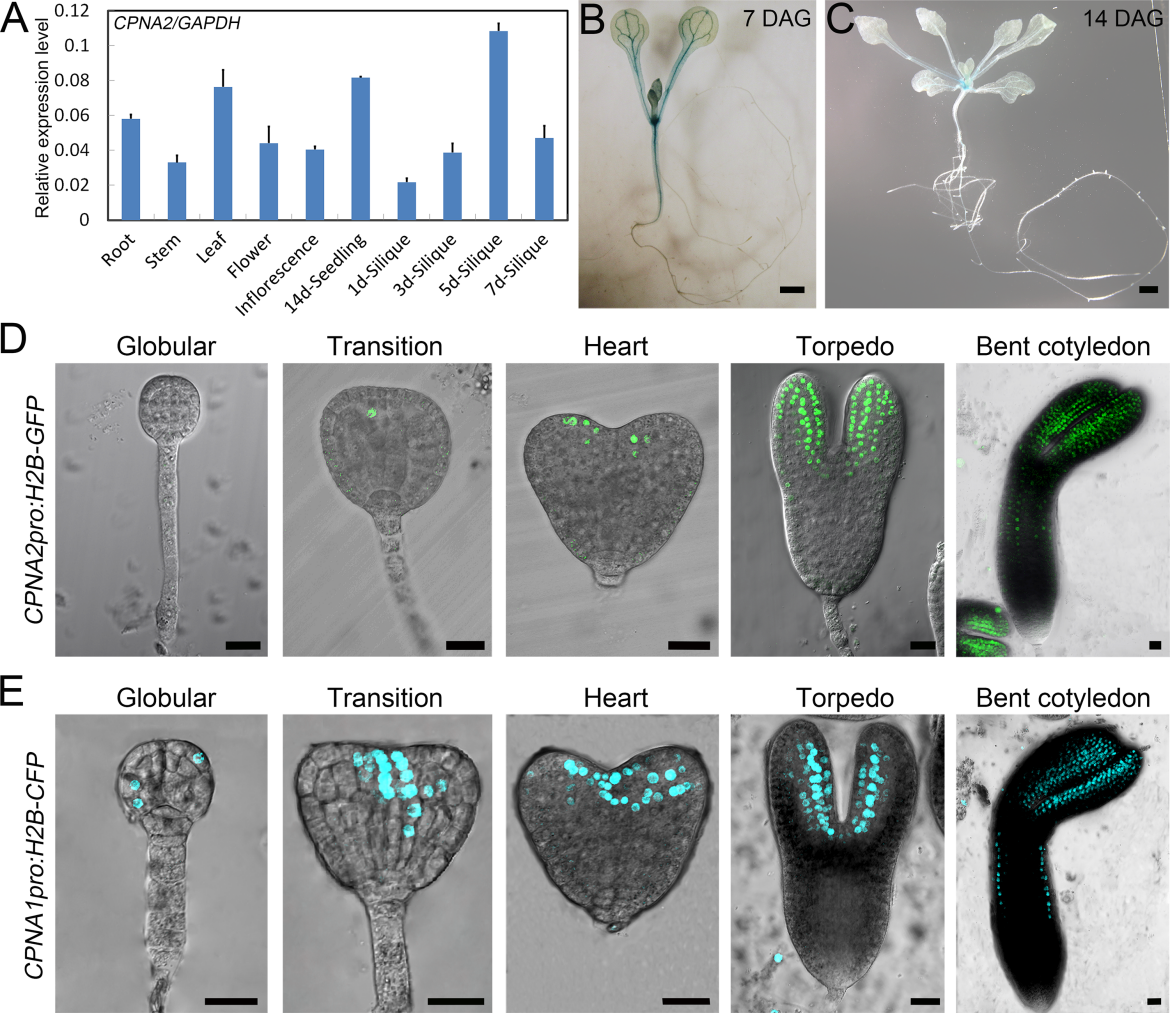
Expression pattern analysis of *CPNA2* and *CPNA1*. (A) qRT-PCR analysis of the transcript levels of *CPNA2* in various tissues. *GAPDH* was used as the control gene. Error bars indicate standard deviation (SD) of three biological replicates. (B and C) GUS staining signals in *CPNA2pro*:*GUS* transgenic seedlings. (B) 7 DAG seedlings. (C) 14 DAG seedlings. Bars = 1 mm. (D) GFP signals in embryos of *CPNA2pro*:*H2B-GFP* transgenic plants. Bars = 20 μm. (E) CFP signals in embryos of *CPNA1pro*:*H2B-CFP* transgenic plants. Bars = 20 μm.

It had been reported that *CPNA2* has an extremely low signal in all tissues and developmental stages using the Genevestigator program, and the CPNA2 protein could not be detected in proteomics studies [14,30]. To further investigate the expression pattern of *CPNA2*, we examined *CPNA2* transcript levels in different *Arabidopsis* tissues using quantitative real-time PCR (qRT-PCR). The result demonstrated that *CPNA2* is expressed in all tissues and is especially highly expressed in the 5 DAP (day after pollination) siliques (Fig 3A). Then, we performed a GUS assay in the transgenic plants carrying the *CPNA2pro*:*GUS* construct, and observed a very strong signal in the SAM and a weaker signal in vascular bundles of 7 DAG (day after germination) seedlings, which largely declined in 14 DAG seedlings (Fig 3B and 3C). No signal was found in mature leaves, flowers, inflorescences and siliques, possibly due to low abundance and dispersion of GUS signals.

To investigate the expression pattern of *CPNA2* during embryo development, we obtained transgenic plants carrying the *CPNA2pro*:*H2B-GFP* construct, and observed the fluorescent signal in the dissected embryos. No GFP (green fluorescent protein) signal was detected in the globular embryos, but GFP signals began to sporadically appear in the transition stage embryos (Fig 3D). When the embryos reached the heart stage, fluorescent signals were located on the adaxial sides of cotyledons (Fig 3D). Next, the signals were mainly detected in cotyledons of the torpedo and cotyledon stages, and were still strongest on the adaxial sides of cotyledons (Fig 3D). Together, these results showed that *CPNA2* is highly expressed in the SAM of early seedlings and embryonic cotyledons.

### Functional divergence of *CPNA2* and *CPNA1* occurs during *Arabidopsis* embryo development

Hill and Hemmingsen reported that *CPNA2* is the paralog of *CPNA1* [15], thus it is possible that they have redundant functions. However, a *CPNA1* mutant, *slp* (*schlepperless*), showed an embryo-defective phenotype that mainly appeared at the heart stage and thereafter, which is different from the *cpna2* mutants [16]. To confirm the previous study, we obtained another *AtCpn60α1* mutant (*cpna1*, SALK_006606) (S1A Fig), and then observed embryo development in the siliques of *cpna1*/+. As expected, nearly a quarter of the examined embryos (25.71%, n = 579) showed the abnormal phenotype. The abnormal embryos had highly reduced cotyledons, a larger angle between cotyledons, and developed more slowly from the torpedo stage (S1B Fig), which was similar to the phenotype of the *slp* embryos.

As shown above, *cpna1* and *cpna2* mutants had very different embryo-defective phenotypes, which could be caused by functional divergence and/or different expression patterns of the two genes. To investigate the expression pattern of *CPNA1* during embryo development, we obtained transgenic plants carrying the *CPNA1pro*:*H2B-CFP* construct, and observed CFP (cyan fluorescent protein) signals in the dissected embryos. The fluorescent signal was originally detected in protoderm cells at the globular stage, and then concentrated in the SAM at the transition stage (Fig 3E). When the embryos developed into heart, torpedo, and cotyledon stages, most CFP signals were specifically redistributed on the adaxial sides of cotyledons (Fig 3E).

As the above results demonstrated, *CPNA1* has a similar expression pattern to *CPNA2* during embryo development, while it is expressed more widely. This indicates that the different embryo-defective phenotypes of the *cpna1* and *cpna2* mutants are likely to be caused by functional divergence of the two genes, but not due to differences in expression pattern. Moreover, these findings also suggested that *CPNA1* mainly plays a role at the torpedo stage and thereafter, whereas *CPNA2* is crucial to reach the heart stage for *Arabidopsis* embryos.

### CPNA2 and CPNA1 have specific AtCpn60β subunits as their functional partners

Ch-Cpn60s had been considered to be hetero-oligomers consisting of equal amounts of Cpn60α and Cpn60β, based on several studies conducted in *Pisum sativum*, *Brassica napus*, *Arabidopsis thaliana*, and *Spinacia oleracea* [11, 12, 13, 31]. Moreover, because CPNA2 and CPNA1 could play different roles during embryo development, we wondered which AtCpn60β subunits could interact with CPNA2 or CPNA1 to form specific chaperonins. Using AtPID (*Arabidopsis thaliana* Protein Interactome Database) [32], we first predicted the functional partners of CPNA2 and CPNA1. The result showed that AtCpn60β1 and AtCpn60β2 had much higher scores than AtCpn60β3 and AtCpn60β4 among the predicted functional partners of CPNA1 (S2 Table). In contrast, AtCpn60β3 and AtCpn60β2 were the top two predicted functional partners of CPNA2, and AtCpn60β3 had a far higher score than the other candidates (S2 Table). These results implied that CPNA2 and CPNA1 could possibly interact with different AtCpn60β subunits to form specific functional chaperonins.

To further clarify the functional partners of CPNA2 and CPNA1, we obtained T-DNA insertion mutants of *AtCpn60β1* (*CPNB1*), *AtCpn60β2* (*CPNB2*), *AtCpn60β3* (*CPNB3*), and *AtCpn60β4* (*CPNB4*) from ABRC (S2A Fig). Homozygous mutant plants of all *AtCpn60β* genes could be obtained and had normal fertility. Through reverse transcription PCR (RT-PCR) analysis, we also confirmed the complete loss of the corresponding transcripts in the *cpnb2*, *cpnb3* and *cpnb4* mutants, and the enormous reduction of the *CPNB1* transcript in the *cpnb1* mutant (S2B Fig). We then crossed these mutants pairwise to obtain double heterozygous plants. In all double heterozygous plants, we only observed aberrant seeds in siliques of the *cpnb1*/+ *cpnb2*/+ and *cpnb2*/+ *cpnb3*/+ plants (S2C Fig), implying that the *cpnb1 cpnb2* and *cpnb2 cpnb3* double homozygous embryos were possibly abnormal.

**Fig 4:**
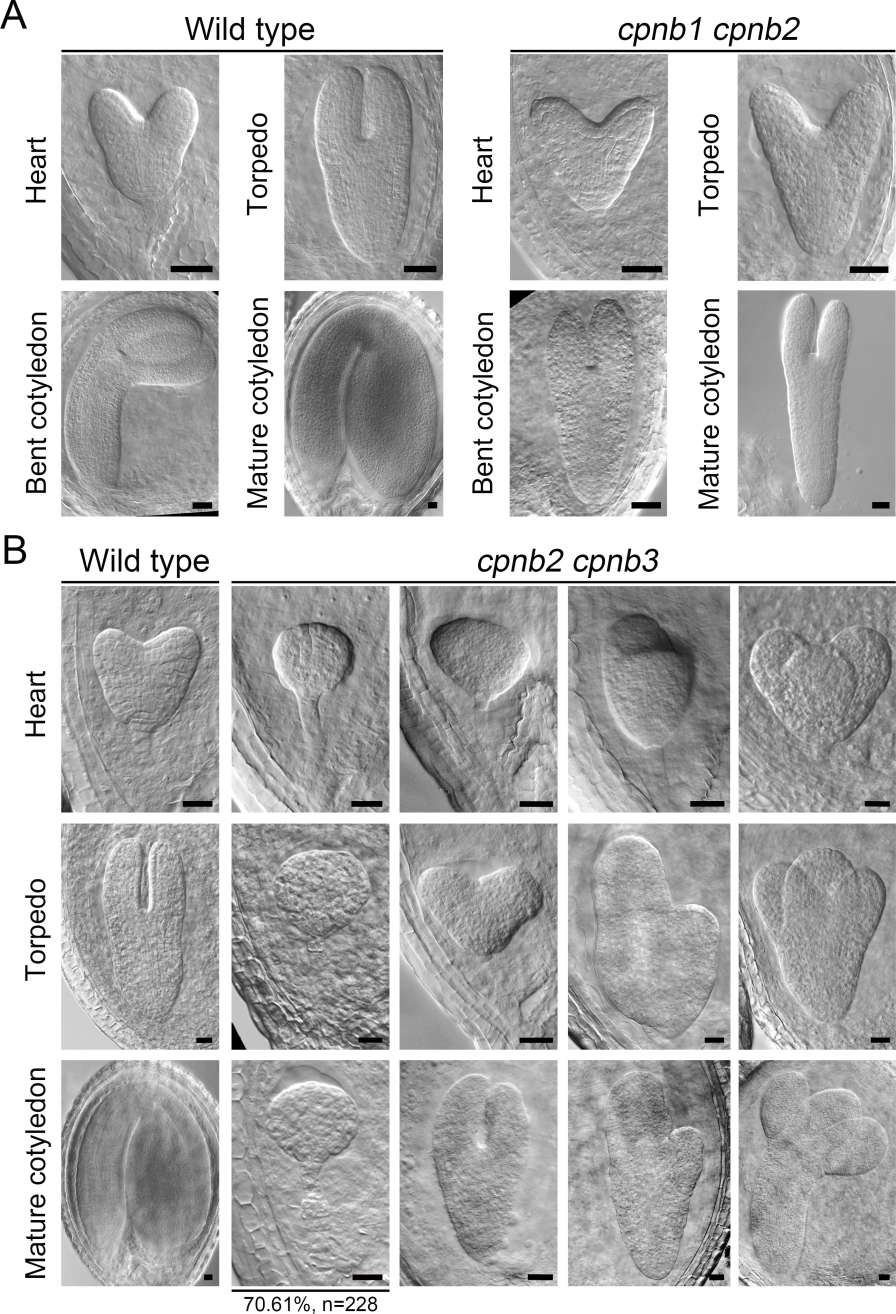
Phenotypic observation of *cpnb1 cpnb2* and *cpnb2 cpnb3* embryos. (A) Phenotypic observation of embryos from wild-type plants and embryos in abnormal ovules from *cpnb1*/+ *cpnb2* plants. (B) Phenotypic observation of embryos from wild-type plants and embryos in abnormal ovules from *cpnb2 cpnb3*/+ plants. Bars = 20 μm.

We also obtained *cpnb1*/+ *cpnb2* and *cpnb2 cpnb3*/+ plants in the self-crossed progenies of the *cpnb1*/+ *cpnb2*/+ and *cpnb2*/+ *cpnb3*/+ mutants, respectively. As expected, siliques of the *cpnb1*/+ *cpnb2* and *cpnb2 cpnb3*/+ plants contained numerous aberrant seeds, at a frequency of 25.43% (n = 1050) and 25.64% (n = 1166), respectively, suggesting that the *cpnb1 cpnb2* and *cpnb2 cpnb3* embryos were probably abnormal. To clarify the cause for the abnormality of the *cpnb1 cpnb2* and *cpnb2 cpnb3* embryos, we examined the developmental processes of these double homozygous embryos in the aberrant seeds. In siliques of the *cpnb1*/+ *cpnb2* plants, the *cpnb1 cpnb2* embryos had a similar shape to wild-type embryos at the heart stage, apart from slightly smaller cotyledons and a larger angle between cotyledons. However, the *cpnb1 cpnb2* embryos began to display highly reduced cotyledons at the torpedo stage and thereafter compared to the wild-type embryos (Fig 4A). In contrast, we observed that the majority of the *cpnb2 cpnb3* embryos (70.61%, n = 228) were arrested at the globular stage in the siliques of the *cpnb2 cpnb3*/+ plants, while the other *cpnb2 cpnb3* embryos showed various phenotypes, including remarkable retardation of embryo development, and variation of cotyledon shape and number (Fig 4B).

Overall, we found that *cpnb1 cpnb2* embryos had a very similar phenotype to *cpna1* embryos, echoing the previous study that showed that *cpnb1 cpnb2* seedlings were analogous to *cpna1* seedlings [20]. In addition, although the penetrance was incomplete possibly due to partial complement of *CPNB1*, the majority of the *cpnb2 cpnb3* embryos were shown to phenocopy the *cpna2* embryos. Combined with the prediction from AtPID (S2 Table), this result further suggested that CPNA1 is likely to form a functional chaperonin with CPNB1 and CPNB2 during *Arabidopsis* embryo development, whereas CPNB2 and CPNB3 were the functional partners of CPNA2. Moreover, since the *cpnb1*/+ *cpnb3*/+ plants showed normal seed development, it seemed that CPNB2 was a versatile AtCpn60β subunit which could form functional chaperonins with both CPNA1 and CPNA2, and was sufficient to support embryo development alone.

### KASI is a specific substrate of the functional chaperonin containing CPNA2

As the previous results demonstrated, we found that CPNA2 and CPNA1 have nonredundant functions during embryo development. Moreover, it had been widely reported that some chaperonins containing specific subunits had unique substrates and played an important role under certain circumstances [21, 33, 34, 35]. Based on these results, it is possible that the chaperonin containing CPNA2 has some specific substrates that cannot be folded by the chaperonin containing CPNA1, thus affecting the specific developmental process of *Arabidopsis* embryos.

**Fig 5:**
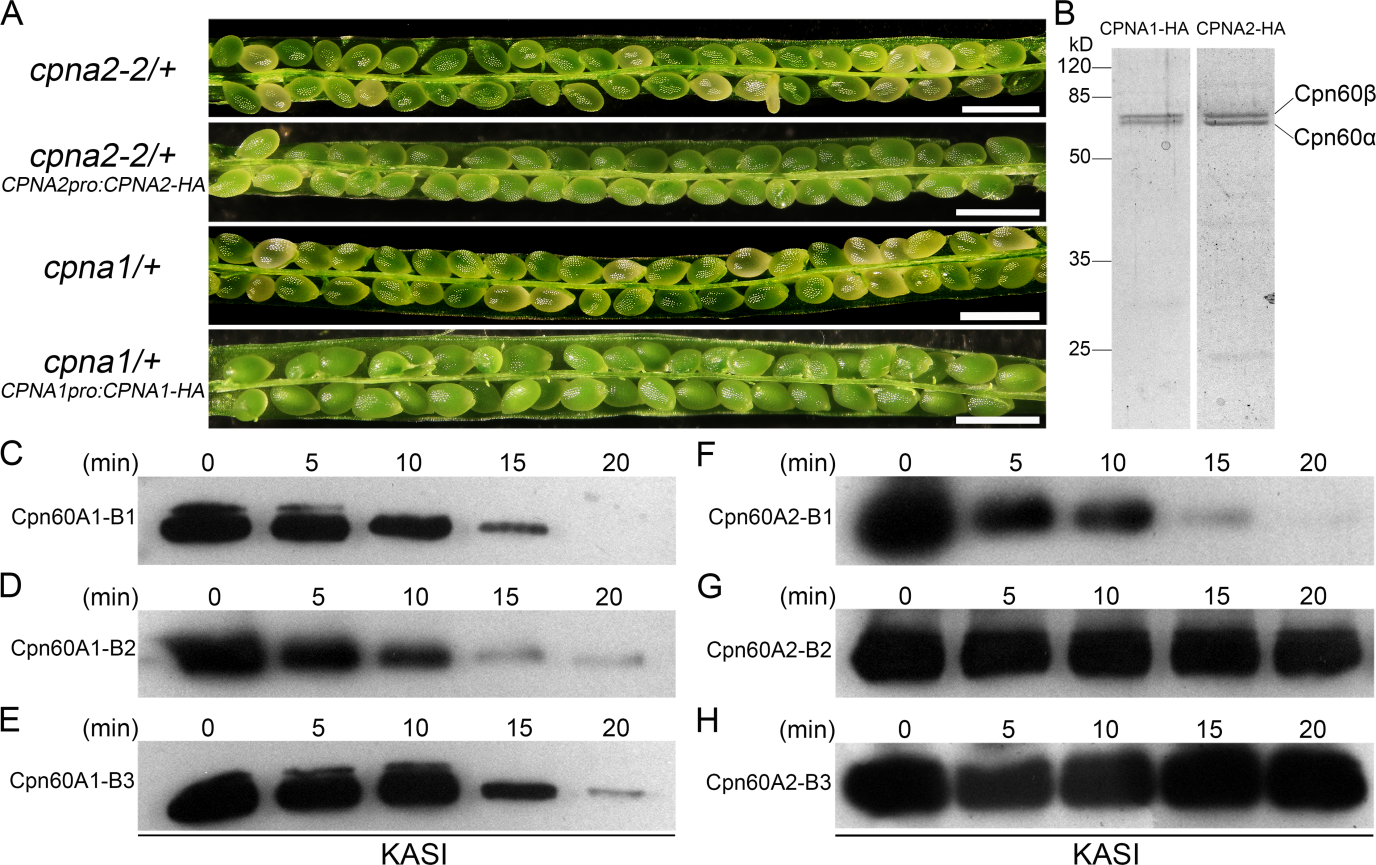
Co-IP assay using HA-tagged Cpn60α subunits and proteinase K protection assay of KASI. (A) Silique phenotypes of *cpna2-2*/+ complemented with *CPNA2pro*:*CPNA2-HA* and *cpna1*/+ complemented with *CPNA1pro*:*CPNA1-HA*. Bars = 1 mm. (B) SDS-PAGE of the CPNA2 and CPNA1 immunoprecipitation fractions. The total immunoprecipitates were separated on 12% SDS-PAGE gels and stained with Coomassie Brilliant Blue (CBB). (C-H) Immunoblotting analysis of KASI after proteinase K treatments in the presence of Cpn60A1-B1 (C), Cpn60A1-B2 (D), Cpn60A1-B3 (E), Cpn60A2-B1 (F), Cpn60A2-B2 (G) and Cpn60A2-B3 (H). Each experiment was repeated at least three times with comparable results.

To examine this possibility, we first introduced two chimeric genes encoding an HA (influenza hemagglutinin protein epitope) tag fused to the C-terminus of CPNA2 or CPNA1 into *cpna2-2*/+ and *cpna1*/+ mutants, respectively. The *CPNA2pro*:*CPNA2-HA* and *CPNA1pro*:*CPNA1-HA* constructs fully restored the fertility of *cpna2-2*/+ and *cpna1*/+ plants, respectively (Fig 5A), indicating that HA-tag has no effect on the functions of CPNA2 and CPNA1. Since expression of the *CPNA2* gene in vegetative tissues is very low [14], we also obtained transgenic lines carrying the *35Spro*:*CPNA2-HA* or *35Spro*:*CPNA1-HA* construct, and conducted Co-IP assay in 7 DAG transgenic and wild-type seedlings using the μMACS HA isolation kit (Miltenyi Biotec). The immunoprecipitates were separated by SDS-PAGE and then analyzed by liquid chromatography-tandem mass spectrometry (LC-MS/MS) (Fig 5B). The MS analysis showed that KASI, a protein involved in *de novo* fatty acid synthesis, had more peptides and much higher scores in CPNA2 immunoprecipitation fractions than in CPNA1 and WT immunoprecipitation fractions (Table 1 and S1 Data). These results suggested that KASI was a possible specific substrate of the chaperonin containing CPNA2. Moreover, the MS data also showed that CPNB3 was not detected in CPNA1 immunoprecipitation fractions, but had high scores in CPNA2 immunoprecipitation fractions (Table 1), echoing the previous genetic results demonstrating that CPNB3 is a functional partner of CPNA2 but not CPNA1.

**Table 1:** Summary of chaperonin subunits and KASI detected in CPNA2 and CPNA1 immunoprecipitation fractions.

Name	CPNA2-Replicate1			CPNA2-Replicate2			CPNA1-Replicate1			CPNA1-Replicate2		
	PSMs^a^	Coverage(%)	Score^b^	PSMs	Coverage(%)	Score	PSMs	Coverage(%)	Score	PSMs	Coverage(%)	Score
CPNA1	116	71.67	355.18	78	61.26	284.28	72	47.27	183.28	50	46.42	163.21
CPNA2	114	62.26	293.35	78	54.26	242.19	ND^c^	ND	ND	4	7.83	10.81
CPNB1	222	76.67	586.73	131	74.50	466.98	119	52.50	316.91	34	42.33	111
CPNB2	213	74.16	563.29	129	74.83	460.51	127	52.18	303.82	28	37.25	89.63
CPNB3	98	64.99	213.15	57	46.03	180.23	ND	ND	ND	ND	ND	ND
CPNB4	14	25.04	21.61	4	7.53	9.41	13	4.75	25.49	ND	ND	ND
KASI	15	37.84	36.53	6	11.25	11.54	1	2.45	1.77	1	2.86	2.17

^a^Peptide-Spectrum Match.

^b^Scores are obtained by SEQUEST HT.

^c^Not detected.

To further confirm that KASI is a specific substrate of the chaperonin containing CPNA2, we also performed a proteinase K protection assay. This assay takes advantage of the formation of a highly stable *cis*-ternary complex consisting of substrate, chaperonin and co-chaperonin in the presence of ADP [36, 37]. This *cis*-ternary complex could sequester a substrate into the cavity of a chaperonin, thus protecting the substrate from digestion by proteinase K. Recombinant CPNA1, CPNA2, CPNB1, CPNB2, CPNB3, Cpn20 (a co-chaperonin subunit in *Arabidopsis*), and KASI proteins were overexpressed in *Escherichia coli* and then purified on Ni-NTA agarose resin. Subsequently, the various chaperonins consisting of AtCpn60α and AtCpn60β subunits were reconstituted according to previously described method [31]. After purification by gel-filtration chromatography, the reconstituted chaperonins were used in proteinase K protection assays of denatured KASI protein. As shown in Fig 5C, 5D and 5E, KASI was digested by proteinase K in the presence of Cpn60A1-B1, Cpn60A1-B2 or Cpn60A1-B3, indicating that KASI was not likely to form stable *cis*-ternary complexes with Cpn20 and chaperonins containing CPNA1. In contrast, in the presence of Cpn60A2-B2 or Cpn60A2-B3, almost all of the KASI protein was protected from digestion by proteinase K (Fig 5G and 5H), which was likely due to the formation of the stable *cis*-ternary complexes. Taken together, these results showed that denatured KASI protein could only be captured and refolded by Cpn60A2-B2 and Cpn60A2-B3, but not by chaperonins containing CPNA1, confirming that KASI is a specific substrate of the functional chaperonins containing CPNA2.

In addition, as shown in Table 1, CPNB1 had very high scores in both CPNA2 immunoprecipitation fractions, indicating that CPNB1 could likely interact with CPNA2. To know whether CPNB1 was a functional partner of CPNA2, we also reconstituted the chaperonin consisting of CPNA2 and CPNB1 (Cpn60A2-B1) and performed a proteinase K protection assay of denatured KASI using Cpn60A2-B1. As shown in Fig 5F, KASI was not protected from digestion by proteinase K in the presence of Cpn60A2-B1, indicating that Cpn60A2-B1 was not a functional chaperonin that can assist the folding of KASI. This result showed that although CPNB1 could interact with CPNA2 when the CPNA2 protein was overexpressed *in vivo*, it could not form a full-functional chaperonin with CPNA2 alone, providing further evidence supporting the above genetic findings that suggested CPNB1 was not a functional partner of CPNA2.

### Premature arrest of *cpna2* embryos is likely caused by reduction of KASI protein

**Fig 6:**
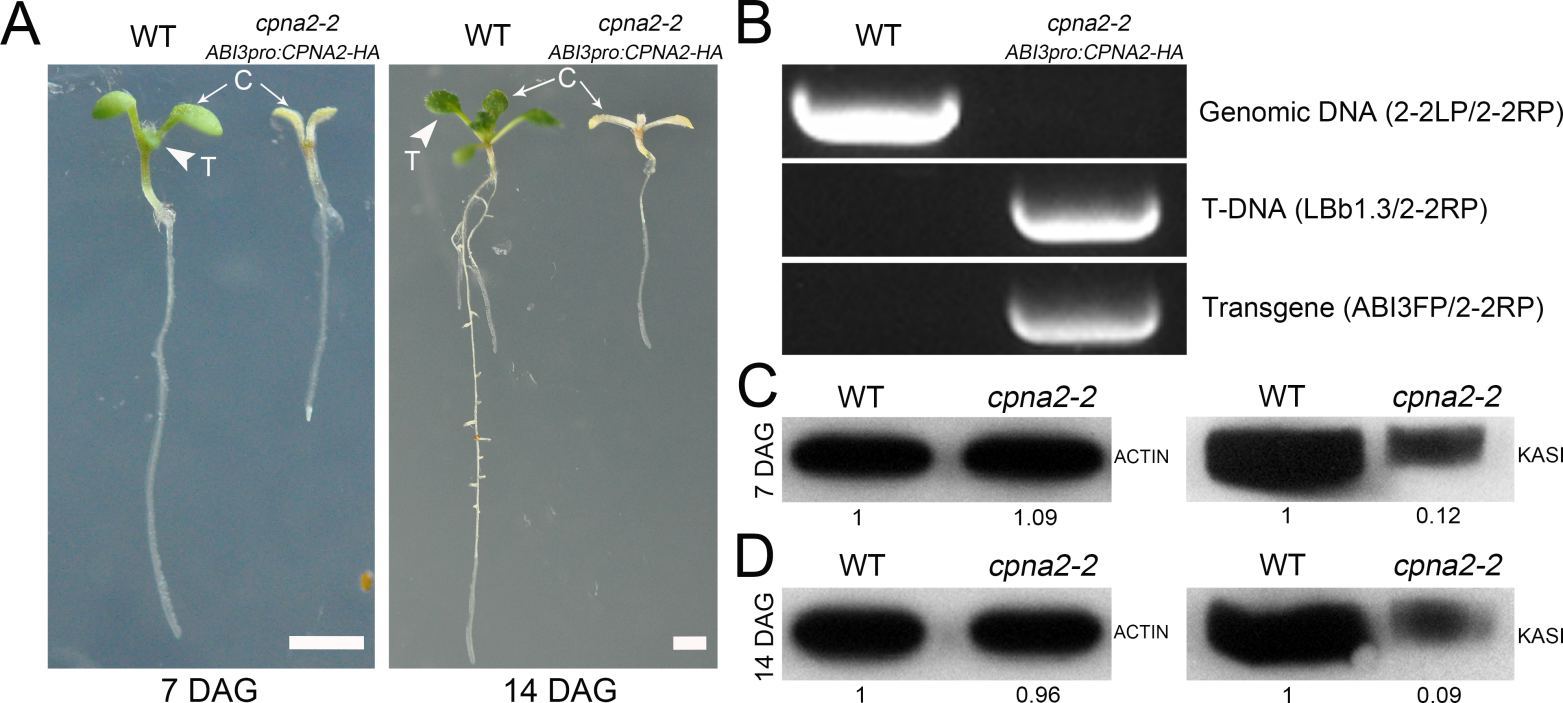
Partial complementation of the *cpna2-2* mutant using an *ABI3pro*:*CPNA2-HA* vector. (A) 7 and 14 DAG seedlings of WT and *cpna2-2* homozygous mutants. Arrows indicate cotyledons, and arrowheads indicate true leaves. C, cotyledon; T, true leaf. Bars = 2 mm. (B) Genotypic analysis of *cpna2-2* homozygous seedlings carrying an *ABI3pro*:*CPNA2-HA* vector. (C and D) Protein levels of KASI in 7 (C) and 14 (D) DAG seedlings of WT and *cpna2-2* homozygous mutants. ACTIN was used as the loading control, and the protein levels of ACTIN and KASI were determined by immunoblotting using the corresponding antibodies. Numbers under lanes indicate the relative band intensities quantified by ImageJ. Each experiment was repeated at least three times with comparable results.

As demonstrated above, KASI is a specific substrate of the functional chaperonin containing CPNA2. To know whether the level of KASI protein was reduced in the *cpna2* homozygous mutant, we first rescued the development of *cpna2-2* homozygous embryos using a *ABI3pro*:*CPNA2-HA* construct. Since the *ABI3* gene is specifically expressed in seeds [38, 39], we obtained *cpna2-2* homozygous seedlings from *ABI3pro*:*CPNA2-HA* transgenic lines (Fig 6A). The abnormal seedlings had the white cotyledons and could not develop true leaves even on the fourteenth day after germination (Fig 6A). Genotypic analysis of the abnormal seedlings also confirmed that they were *cpna2-2* homozygous mutants partially rescued by the *ABI3pro*:*CPNA2-HA* construct (Fig 5B). Then we examined KASI protein levels in 7 and 14 DAG seedlings of WT and *cpna2-2* by immunoblotting. As shown in Fig 6C and 6D, KASI protein levels in 7 and 14 DAG seedlings of *cpna2-2* were reduced to approximately one-tenth of the level in contemporaneous WT seedlings. This result indicated that the KASI protein level could be largely reduced due to loss-of-function of *CPNA2 in vivo*.

**Fig 7:**
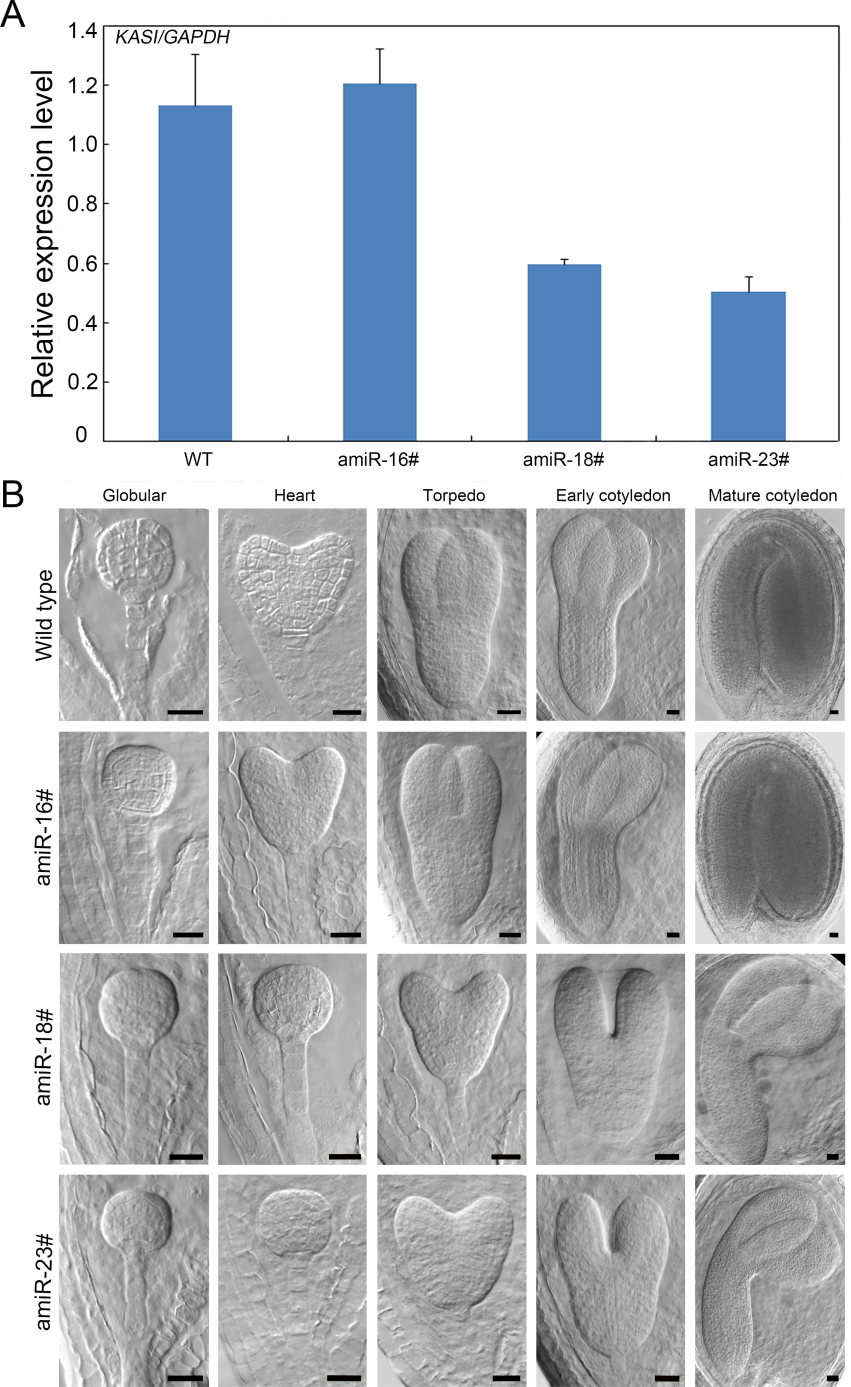
Detection of the expression levels of *KASI* and phenotypic observation of embryos in *CPNA2pro*:*amiR-KASI* transgenic lines. (A) qRT-PCR analysis of the transcript levels of *KASI* in 10 DAG seedlings of wild type, amiR-16#, amiR-18# and amiR-23# lines. *GAPDH* was used as the control gene. Error bars indicate SD of three biological replicates. (B) Phenotypic observation of the embryos in wild type and lines 16, 18, 23 of *CPNA2pro*:*amiR-KASI* transgenic plants. Bars = 20 μm.

In addition, Wu and Xue reported that *KASI* was crucial for embryo development and *KASI* deficiency resulted in disrupted embryo development before the globular stage [40]. Therefore, it is possible that the decline of KASI protein level in *cpna2* embryos causes abortion of the mutant embryos. To examine this possibility, we constructed a *CPNA2pro*:*amiR-KASI* vector to specifically reduce the expression level of *KASI* in the embryos of transgenic lines at the transition stage and thereafter. In T1 generation transgenic plants, we chose three lines for follow-up studies. As shown in Fig 7A, we found that the expression levels of *KASI* in lines 18 and 23 were reduced by almost half, whereas the expression level of *KASI* in line 16 remained unchanged. Then we carefully examined embryo development in ovules of wild type, line 16, line 18, and line 23. In the ovules of line 16, embryos developed similarly to wild type (Fig 7B). In contrast, when wild-type embryos reached the heart stage, almost all the embryos of line 18 and line 23 still stayed at the globular stage, and reached the heart stage when normal embryos entered the torpedo stage (Fig 7B). Embryos in the ovules of line 18 and line 23 ultimately could reach the cotyledon stage (Fig 7B), and the fertility of line 18 and line 23 plants was not affected. Moreover, we also counted the percentages of all embryonic morphologies in the 3, 4, 5, and 7 DAP siliques of wild type and transgenic lines (S3 Table), further confirming the delayed embryo development in lines 18 and 23, consistent with the morphological observations.

By observing the embryo development of the *KASI* knock-down transgenic lines, we found that reduction of the expression level of *KASI* clearly delayed the process by which globular embryos develop into heart-shaped embryos. This finding suggested that the KASI level is crucial to reach the heart stage for *Arabidopsis* embryos, implying that abnormality of the *cpna2* embryos is likely to be caused by a decrease of correctly folded KASI protein in the mutant embryos.

### Evolutionary relationship of Cpn60α1 and Cpn60α2

To investigate the evolutionary relationship of Cpn60α1 and Cpn60α2, we obtained protein sequences of *CPNA1* and *CPNA2*, excluding the transit peptides predicted by TargetP [41], and then searched for homologous proteins in various species using BLAST (Basic Local Alignment Search Tool, http://blast.ncbi.nlm.nih.gov/Blast.cgi). By sequence alignment, we constructed the phylogenetic tree of Cpn60α1 and Cpn60α2. The tree showed that Cpn60α1 orthologs exist in monocotyledons, dicotyledons, gymnosperms (*Picea sitchensis*), bryophytes (*Physcomitrella* *patens*) and algae (*Chlamydomonas reinhardtii*), whereas Cpn60α2 orthologs form a separate cluster and only exist in monocotyledons and dicotyledons (S3 Fig). The result suggested that *Cpn60α1* is more primitive than *Cpn60α2*, and *Cpn60α2* probably originated from gene duplication and variation of *Cpn60α1* in angiosperms.

### Structural basis of functional specialization of CPNA2

**Fig 8:**
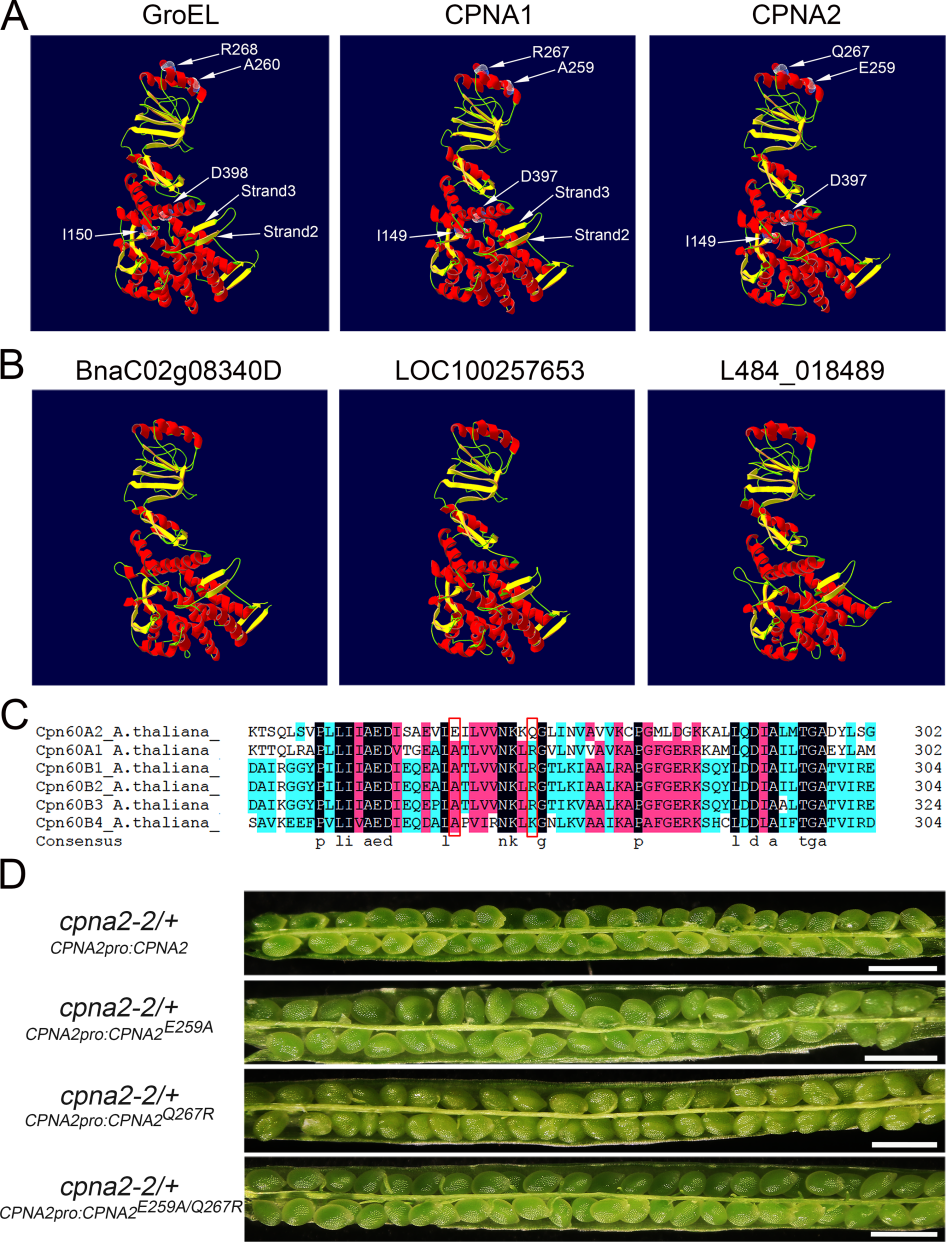
Structural analysis of the CPNA2 protein. (A) 3D structural models of GroEL, CPNA1, and CPNA2. The predicted structural models of CPNA1 and CPNA2 were generated with SWISS-MODEL, and the structural model of GroEL (PDB 1AON, Chain A) was used as the template. (B) 3D structural models of BnaC02g08340D, LOC100257653, and L484_018489. BnaC02g08340D, LOC100257653 and L484_018489 are the orthologs of CPNA2 in *Brassica napus*, *Vitis vinifera* and *Morus notabilis*, respectively. The structural model of GroEL (PDB 1AON, Chain A) was used as the template. (C) Sequence alignment of the chaperonin subunits in *Arabidopsis*. Amino acid residues in position 259 and 267 are included in the red frames. Sequences were aligned in DNAMAN 6.0. (D) Complementation of the *cpna2-2*/+ plants with the *CPNA2pro*:*CPNA2*, *CPNA2pro*:*CPNA2*^*E259A*^, *CPNA2pro*:*CPNA2*^*Q267R*^ or *CPNA2pro*:*CPNA2*^*E259A/Q267R*^ construct. Bars = 1 mm.

As the above results showed, the functional chaperonin containing CPNA2 could specifically assist in the folding of KASI and could play a unique role during *Arabidopsis* embryo development. To clarify the structural basis of functional specialization of CPNA2, we first analyzed the three-dimensional (3-D) structures of GroEL, CPNA1, and CPNA2 through homology modeling. As shown in Fig 8A, compared with GroEL and CPNA1, CPNA2 lacked strands 2 and 3. To confirm the importance of strands 2 and 3, we also did homology modeling of the CPNA2 orthologs in *Brassica napus*, *Vitis vinifera*, and *Morus notabilis*. Surprisingly, these orthologs all possess strands 2 and 3 (Fig 8B), suggesting that the lack of strands 2 and 3 is not likely to be necessary for functional specialization of CPNA2. Moreover, Ile 150 (I150) and Asp 398 (D398), which are crucial for ATP/ADP binding in GroEL, are also highly conserved in CPNA1 and CPNA2 (Fig 8A).

In previous studies, a few positions in the apical domain of GroEL had been proposed as substrate binding sites, which are highly conserved in many species [42–44]. To know whether these positions led to functional specialization of CPNA2, we first examined the corresponding positions in the homologous proteins of CPNA1 and CPNA2 by protein sequence alignment (S4 Fig). The result showed that the highly conserved hydrophobic residue Ala 259 in the orthologs of CPNA1 is converted to a hydrophilic residue Glu or Ser in the orthologs of CPNA2, while the conserved positively charged residue Arg 267 in Cpn60α1 is mostly replaced by an uncharged residue Gln or Asn in Cpn60α2 (S4 Fig). Furthermore, Glu 259 and Gln 267 of the CPNA2 protein are also unique in all the *Arabidopsis* ch-Cpn60 subunits (Fig 8C), implying that the two positions are likely the cause for functional specialization of CPNA2. To confirm this conjecture, Glu 259 and Gln 267 of CPNA2 were converted to Ala 259 and Arg 267, respectively, through site-directed mutagenesis, and then a *CPNA2pro*:*CPNA2*, *CPNA2pro*:*CPNA2*^*E259A*^, *CPNA2pro*:*CPNA2*^*Q267R*^ or *CPNA2pro*:*CPNA2*^*E259A/Q267R*^ construct was introduced into *cpna2-2*/+ plants. Unexpectedly, *cpna2-2*/+ plants carrying the *CPNA2pro*:*CPNA2*, *CPNA2pro*:*CPNA2*^*E259A*^, *CPNA2pro*:*CPNA2*^*Q267R*^ or *CPNA2pro*:*CPNA2*^*E259A/Q267R*^ construct all had normal fertility (Fig 8D), suggesting that the two conserved residues in the orthologs of CPNA2 are not crucial for the functional specialization of CPNA2. These results indicated that CPNA2 is likely to utilize a few new positions to bind KASI, and that detailed structural information of the chaperonin containing CPNA2 is required to further elucidate the mechanism of KASI folding.

## Discussion

### Different Cpn60α subunits have specific Cpn60β subunits as their functional partners in *Arabidopsis*

Chloroplast chaperonins are composed of two types of chaperonin subunits, Cpn60α and Cpn60β, which is different from the chaperonins in bacteria and mitochondria. Several previous studies demonstrated that ch-Cpn60s consisting of nearly equal amounts of Cpn60α and Cpn60β were the native form of chloroplast chaperonins *in vivo*, although the Cpn60β subunit could also form the chaperonin complex in reconstitution experiments alone [11, 12, 13, 31]. Among the four Cpn60β subunits in *Arabidopsis*, AtCpn60β1, AtCpn60β2, and AtCpn60β3 have more than 90% identity, while AtCpn60β4 shares only 60% identity with the other three AtCpn60β subunits. However, the homo-oligomers reconstituted with AtCpn60β1, AtCpn60β2 or AtCpn60β3 have unique physicochemical properties, different preferences for various co-chaperonins, and distinct abilities of folding substrates, implying the functional divergence of Cpn60β1/2/3 in *Arabidopsis* [45].

In this study, we analyzed the phenotypes of different combinations of *AtCpn60β* double mutants, and found that *cpnb1 cpnb2* and *cpnb2 cpnb3* double mutant embryos phenocopied *cpna1* and *cpna2* embryos, respectively. This finding suggested that CPNA1 plays a role in embryo development together with CPNB1 and CPNB2, while CPNA2 can function with CPNB2 and CPNB3. Moreover, we found that CPNB3 could not be detected in the CPNA1 immunoprecipitation fractions, whereas it was abundant in the CPNA2 immunoprecipitation fractions (Table 1). This result indicated that CPNA1 had far lower affinity for CPNB3 compared with CPNA2, further confirming that CPNB3 is the functional partner of CPNA2 but not CPNA1, consistent with the genetic results. In addition, we found that the chaperonin complex reconstituted with CPNA2 and CPNB1 could not protect KASI from proteinase K (Fig 5F), thus suggesting that CPNB1 is the functional partner of CPNA1 but not CPNA2, consistent with the genetic results. These findings provided evidence that different AtCpn60α subunits could bind specific AtCpn60β subunits as their functional partners, indicating the functional divergence of Cpn60α subunits in *Arabidopsis*.

Moreover, we also found that although CPNB1 and CPNA2 do not appear to function together in the folding of KASI, CPNB1 was abundant in the CPNA2 immunoprecipitation fractions (Table 1). It was also reported that AtCpn60β1/2/3 and AtCpn60α1 usually formed the native chaperonin together *in vivo* [21, 30], even though we did not detect CPNB3 in CPNA1 immunoprecipitation fractions possibly due to low affinity of CPNB3 for CPNA1. These results suggested that all the AtCpn60β1/2/3 subunits are usually mixed into native chaperonins containing the Cpn60α subunit *in vivo*, although they have different affinity for specific Cpn60α subunit. Moreover, although it had been reported that the native chaperonin containing Cpn60β4 in *Arabidopsis* is composed of seven Cpn60α1, two Cpn60β4, and five Cpn60β1/2/3 [21], the exact proportions of AtCpn60β1, AtCpn60β2, and AtCpn60β3 in native chaperonins are difficult to determine due to similar molecular weights and high identity of AtCpn60β1/2/3. Further study is still needed to clarify the stoichiometry of subunits in native ch-Cpn60s of *Arabidopsis*.

### Functional chaperonins containing CPNA2 can specifically assist in the folding of KASI

Although multiple chaperonin genes are present in a high proportion of prokaryotes and eukaryotes, the biological significance of duplication and variation of chaperonin genes has yet to be fully elucidated. Recently, a study on the type II chaperonin of *Sulfolobales* showed that three different chaperonin subunits (α, β, γ) could form three types of chaperonins at different temperatures, and specific chaperonins could fold a distinct range of substrates to adapt to environmental changes [33]. Moreover, it was also reported that GroEL1 in *Mycobacterium smegmatis* specifically interacts with KasA (a key component of type II Fatty Acid Synthesis) to affect mycolic acid synthesis and biofilm formation, whereas GroEL2 provides the housekeeping chaperone function [35]. In the field of chloroplast chaperonins, Zhang and coworkers recently determined the crystal structure of the apical domains of Cpn60α and Cpn60β1 in *Chlamydomonas reinhardtii*, and elucidated the structural basis for why Cpn60α and Cpn60β subunits have different affinity for substrates and co-chaperonins [46]. Additionally, in line with the divergence of protein sequence, the Cpn60β4 subunit in *Arabidopsis* has a unique structure and the chaperonin containing Cpn60β4 could specifically assist the folding of NdhH [21]. These findings revealed that duplication and variation of chaperonin genes could extend the function of chaperonins in various species.

Here, we found that KASI, a protein involved in *de novo* fatty acid synthesis, was far more abundant in CPNA2 immunoprecipitation fractions than in CPNA1 immunoprecipitation fractions, implying that KASI was likely to be a specific substrate of the chaperonin containing CPNA2. To confirm this conjecture, we conducted the proteinase K protection assay of KASI in the presence of different chaperonin complexes. It was shown that both Cpn60A2-B2 and Cpn60A2-B3 could perfectly protect KASI from digestion by proteinase K, whereas all the chaperonins containing CPNA1 could not protect KASI. This result further showed that the functional chaperonins containing CPNA2 could specifically assist in the folding of KASI, suggesting that CPNA2, a minor Cpn60α subunit, has a unique function in *Arabidopsis*. Moreover, we also examined two conserved positions proposed as substrate binding sites in the orthologs of CPNA2, and found that they were not responsible for the functional specialization of CPNA2. Hence, detailed structural analysis of the chaperonin containing CPNA2 is required to further elucidate the mechanism of KASI folding.

Additionally, it was reported that chaperonins in various species had a wide range of substrates [1], therefore we cannot exclude the possibility that the chaperonin containing CPNA2 has other specific substrates in addition to KASI. Moreover, since we conducted Co-IP assay in 7 DAG seedlings but not in embryos due to technology limitations, it is possible that there are some unknown specific substrates of the chaperonin containing CPNA2 that only exist in embryos. The detection of more specific substrates would further contribute to the functional elucidation of *CPNA2* in *Arabidopsis*.

### Reduction of KASI protein level is likely to cause the abortion of *cpna2* embryos

A number of genes involved in *de novo* fatty acid synthesis are essential for early embryo development in *Arabidopsis*. *GURKE*, a gene encoding the acetyl-CoA carboxylase ACC1, is required for partitioning the apical part of globular embryos in *Arabidopsis* [47], and loss-of-function of *CAC1A*, a gene encoding the biotin carboxyl-carrier protein BCCP1, obviously delayed embryo development from the early globular stage [48]. Moreover, *KASI* deficiency was also found to result in arrested development of most *kasI* embryos before the globular stage, and delayed development of few *kasI* embryos [40]. Additionally, in *Arabidopsis* microarray data sets [49], we found that the expression level of *KASI* has a dramatic increase when embryos reach the heart stage, implying that *KASI* is likely to be crucial for the transition of globular embryos to heart-shaped embryos. To confirm this conjecture, we specifically reduced the expression level of *KASI* in the embryos at the transition stage and thereafter by transforming the *CPNA2pro*:*amiR-KASI* vector. In the transgenic lines, we observed that the process of globular embryos reaching the heart stage was delayed, confirming that *KASI* plays an important role in the formation of heart-shaped embryos.

Since *cpna2* embryos are arrested at the globular stage and loss-of-function of *CPNA2* could result in a significant decrease in the KASI protein level that is crucial for the transition of globular embryos to heart-shaped embryos (Figs 1 and 6), the arrest of *cpna2* embryos is likely due to the reduction of the well-folded KASI protein level in mutant embryos. However, we did not find any *CPNA2pro*:*amiR-KASI* transgenic line in which embryo development is arrested at the globular stage, perhaps because it would be difficult to obtain transgenic lines in which the expression of *KASI* is nearly knocked out since *KASI* is an embryo-lethal gene as reported by Wu and Xue [40]. Additionally, as previously mentioned, other unknown specific substrates of the chaperonin containing CPNA2 might exist in *Arabidopsis* embryos, and these substrates are also possible to play an important role in embryo development. Hence the elucidation of why *cpna2* embryos are arrested at the globular stage still needs further research.

### *CPNA2* is crucial for chloroplast biogenesis during *Arabidopsis* embryo and seedling development

During plastid development, proplastids develop highly organized thylakoid membrane to differentiate into mature chloroplasts, and formation of the thylakoid membrane requires coordinated synthesis and assembly of proteins, pigments, and glycerolipids. In chloroplasts, *de novo* fatty acid (FA) synthesis produces 16:0 and 18:0 FAs that are the building blocks of membrane glycerolipid production [50]. Therefore, FA synthesis is crucial for the formation of the thylakoid membrane and for chloroplast biogenesis.

In this study, we found that *CPNA2* deficiency could result in a significant decrease in the KASI protein level (Fig 6). KASI is a key condensing enzyme involved in *de novo* FA synthesis [50], and therefore, it is likely that *CPNA2* deficiency also disrupts FA synthesis in chloroplasts, thus impeding formation of the thylakoid membrane and chloroplast biogenesis. In accordance with this conjecture, we found that abnormal chloroplasts in *cpna2-2* embryos lacked thylakoid membranes and contained a deeply stained mass (Fig 2D and 2E), indicating that chloroplast biogenesis in *cpna2-2* embryos is severely disrupted. Moreover, the result of GUS staining showed that *CPNA2* is highly expressed in the SAM of *Arabidopsis* seedlings (Fig 3B and 3C), implying that *CPNA2* may also play an important role in chloroplast biogenesis in the SAM. This idea was further supported by the result that the *cpna2-2* homozygous seedlings could not develop true leaves (Fig 6A). Taken together, these results suggest that *CPNA2* is crucial for chloroplast biogenesis, and thus affects the developmental processes of *Arabidopsis* embryos and seedlings.

In the process of biological evolution, gene duplication and variation usually extend the function of original genes to adapt to environmental changes. In this study, we found that CPNA2 in *Arabidopsis* belongs to a unique type of Cpn60α subunits that only exist in angiosperms. Functional chaperonins consisting of CPNA2 and specific Cpn60β subunits could specifically assist in the folding of KASI, and play an important role in the transition of globular embryos to heart-shaped embryos in *Arabidopsis*. This neofunctionalization of Cpn60α subunits in *Arabidopsis* provides a novel insight into the significance of multiple *Cpn60α* genes in plants, and reveals the relationship between duplication and functional specialization of chaperonin genes.

## Materials and methods

### Plant materials and growth conditions

The Columbia ecotype of *Arabidopsis thaliana* was used as the wild type in this study. The T-DNA insertion mutants were obtained from ABRC (Arabidopsis Biological Resource Center), including CS76507, SALK_144574 (*cpna2-3*), SALK_006606 (*cpna1*), SAIL_852_B03 (*cpnb1*), SALK_014547 (*cpnb2*), SALK_099972 (*cpnb3*) and SALK_064887 (*cpnb4*). The *cpna2-2* mutant was obtained from the stock CS76507 using thermal asymmetric interlaced PCR [28]. The T-DNA flanking sequences of the mutants were determined by PCR using specific primer of T-DNA left border (LB) and specific genomic primers (LP and RP). All plants were grown in a greenhouse under long-day condition (16 h light/8 h dark) at 22°C.

### Cloning and plant transformation

To construct *CPNA2pro*:*gCPNA2*, the 5000 bp *CPNA2* genomic fragment was amplified from wild-type genome, and then cloned into *pCambia1300* vector (Cambia).

To construct *CPNA2pro*:*GUS*, the promoter of *CPNA2* was amplified and cloned into *pCambia1381Xb* vector (Cambia).

To construct *CPNA2pro*:*H2B-GFP*, *CPNA1pro*:*H2B-CFP*, *35Spro*:*GFP* and *35Spro*:*CPNA2-GFP*, we first obtained the *pC1300-GFP* and *pC1300-CFP* vectors using the operation procedure described by Ren et al. [51]. Then the promoters of *CPNA2* and *CPNA1* were amplified and inserted into the above vectors to produce *CPNA2pro*:*GFP* and *CPNA1pro*:*CFP*, while the 35S promoter was cloned into *pC1300-GFP* to produce *35Spro*:*GFP*. Finally, the *H2B* coding sequence was amplified and cloned into *CPNA2pro*:*GFP* and *CPNA1pro*:*CFP* to obtain *CPNA2pro*:*H2B-GFP* and *CPNA1pro*:*H2B-CFP*, while the *CPNA2* coding sequence was inserted into *35Spro*:*GFP* to obtain *35Spro*:*CPNA2-GFP*.

To construct *35Spro*:*CPNA2-HA*, *35Spro*:*CPNA1-HA*, *CPNA2pro*:*CPNA2-HA*, *ABI3pro*:*CPNA2-HA* and *CPNA1pro*:*CPNA1-HA*, the 35S promoter, the *CPNA2* promoter, the *CPNA1* promoter and *ABI3* promoter were first cloned into *pCambia1300* vector, respectively, to produce *pC1300-35pro*, *pC1300-CPNA2pro*, *pC1300-CPNA1pro* and *pC1300-ABI3pro*. Then the *CPNA2* coding sequence fused to HA tag (CPNA2-HA) and the *CPNA1* coding sequence fused to HA tag (CPNA1-HA) were amplified and cloned into *pC1300-35pro* to produce *35Spro*:*CPNA2-HA* and *35Spro*:*CPNA1-HA*, while CPNA2-HA and CPNA1-HA were cloned into *pC1300-CPNA2pro* and *pC1300-CPNA1pro*, respectively, to produce *CPNA2pro*:*CPNA2-HA* and *CPNA1pro*:*CPNA1-HA*. Moreover, CPNA2-HA was also cloned into *pC1300-ABI3pro* to produce *ABI3pro*:*CPNA2-HA*.

To construct *CPNA2pro*:*amiR-KASI*, we first obtained one amiRNA sequence (TGATGTAATTTACCTCCGCAG) designed for targeting the *KASI* gene using the Web MicroRNA Designer (WMD3; http://wmd3.weigelworld.org/cgi-bin/webapp.cgi) [52]. Then the amiRNA foldback fragment was generated by overlap extension PCR using four specific primers provided by WMD3 and pRS300 vector as a template. Finally, the amiR-KASI foldback fragment was inserted into the *pC1300-CPNA2pro* vector to produce *CPNA2pro*:*amiR-KASI*.

To construct *CPNA2pro*:*CPNA2*, *CPNA2pro*:*CPNA2*^*E291A*^, *CPNA2pro*:*CPNA2*^*Q299R*^ and *CPNA2pro*:*CPNA2*^*E291A/Q299R*^ vectors, *CPNA2*^*E291A*^, *CPNA2*^*Q299R*^ and *CPNA2*^*E291A/Q299R*^ were amplified from the *CPNA2* coding sequence by site-directed mutagenesis using overlap extension PCR. Then these mutant sequences together with the *CPNA2* coding sequence were cloned into the *pC1300-CPNA2pro* vector to obtain *CPNA2pro*:*CPNA2*, *CPNA2pro*:*CPNA2*^*E291A*^, *CPNA2pro*:*CPNA2*^*Q299R*^ and *CPNA2pro*:*CPNA2*^*E291A/Q299R*^.

After sequencing, all the constructs were transformed into *Arabidopsis* plants using the floral dip method [53]. After screened on Murashige and Skoog medium with 10 mg/L hygromycin, positive transformants were identified by PCR and used for subsequent analysis. All the primers for cloning were listed in S4 Table.

### Ovule clearing and embryo observation

Fresh ovules were first dissected from siliques using two needles and cleared with Hoyer’s solution following the protocol described by Yadegari et al. [54]. Then the embryos in the cleared ovules were observed under the Olympus TH4-200 microscope with differential interference contrast (DIC) optics and photographed by a SPOT Xplorer Camera (Diagnostic Instruments).

### Analysis of GUS staining and observation of embryonic fluorescence

GUS staining was conducted according to the method described by He et al. [55]. The various tissues of *CPNA2pro*:*GUS* plants were incubated in GUS solution for 2 to 3 days at 37°C, and then observed by Olympus SZX12 stereomicroscope and photographed with a digital camera (Cool SNAP, RS Photometric).

To observe the fluorescent signals of embryos in the *CPNA2pro*:*H2B-GFP* and *CPNA1pro*:*H2B-CFP* plants, fresh embryos were isolated from ovules through enzymolysis (1% cellulose and 0.8% macerozyme dissolved in 13% mannitol, enzymolysis for 0.5 h at 37°C), mounted in 10% glycerol, and then observed under a confocal microscope (Fluoview1000; Olympus). The images were obtained under EGFP fluorescence channel (excitation, 488 nm; emission, 505–530 nm) and ECFP fluorescence channel (excitation, 440 nm; emission, 505–530 nm).

### Reverse transcription PCR and quantitative real-time PCR

Total RNA of various *Arabidopsis* tissues were extracted using Trizol reagent (Sigma) and then reverse-transcribed into cDNA with a Reverse Transcription System (TOYOBO). The cDNAs of rosette leaves of the *cpnb1-4* homozygous mutants were used as the templates for PCR analysis with the gene-specific primers. qRT-PCR of *CPNA2* was performed using TransStart Top Green qPCR SuperMix (TransGen, China) with a Rotor-Gene 6000 machine (Corbett Research) and the relative expression levels normalized to *GAPDH* were analyzed by the double standard curves method as described previously [56]. qRT-PCR of *KASI* was performed using TransStart Top Green qPCR SuperMix (TransGen, China) with a Bio-rad CFX Connect machine (BIO-RAD) and the relative expression levels normalized to *GAPDH* were analyzed by the comparative C_T_ method as described previously [57,58]. Three biological and three technical replicates of each sample were made for qRT-PCR analysis. Primers used in the experiments were listed in S4 Table.

### Subcellular localization of CPNA2

The mesophyll protoplasts of *35Spro*:*GFP* and *35Spro*:*CPNA2-GFP* transgenic plants were isolated according to the method described previously [59], and then observed under a confocal microscope (Fluoview1000; Olympus). A 488 nm argon ion laser line was used for excitation of GFP and chlorophyll, while 505–530 nm and 650–675 nm emission filters were used for capturing GFP and chlorophyll autofluorescence, respectively.

### Transmission electron microscopy

The wild-type and *cpna2* embryos in 6 DAP siliques of *cpna2-2*/+ plants were fixed, embedded and sectioned as described by Deng et al. [59]. The ultrathin sections were examined and photographed under a transmission electron microscope (Hitachi HT7700).

### Co-IP-MS/MS

Chaperonin-substrate complexes were isolated from the *35Spro*:*CPNA2-HA* and *35Spro*:*CPNA1-HA* transgenic plants with the μMACS HA isolation kit (Miltenyi Biotec) according to the procedure described previously [21]. In brief, intact chloroplasts were first isolated from 7 DAG seedlings of the transformants by the method described previously [60]. Then the freshly isolated chloroplasts were ruptured in lysis buffer (50 mM Tris-HCl pH 8.0, 0.01% Tween 20, 10 mM MgCl_2_, 20 mM glucose, 30 U/ml hexokinase) plus protease inhibitor cocktail (Biotool). After lysis of chloroplasts, ADP (Sigma) was added into the lysates to reach a concentration of 10 mM, and then the lysates were centrifuged at 20,000 g for 10 min. The supernatants were transferred to new tubes and then NaCl was added into the supernatants to reach a final concentration of 150 mM. After incubating with 50 μl anti-HA Microbeads for 2.5 h at 4°C, the mixture was transferred to columns placed in a magnetic field. After rinsing four times with 200 μl washing buffer I (50 mM Tris-HCl pH 8.0, 1% Triton X-100, 0.5% Sodium deoxycholate, 150 mM NaCl, 5 mM ADP), twice with 200 μl washing buffer II (50 mM Tris-HCl pH 8.0, 1% Triton X-100, 150 mM NaCl, 5 mM ADP) and once with washing buffer III (25 mM Tris-HCl pH 7.5, 5 mM ADP), the immunoprecipitates were then eluted with 50 μl elution buffer (50 mM Tris-HCl pH 6.8, 50 mM DTT, 1% SDS, 1 mM EDTA, 0.005% bromophenol blue, 10% glycerol).

After elution, the immunoprecipitates were in-gel digested and analyzed by mass spectrometry as described by Wang et al. [61] with minor modification. In brief, the total protein was loaded to the gel and SDS-PAGE was conducted. After electrophoresis, the gel was stained, sliced and in-gel digested by trypsin, and then the desalted peptides were dissolved in 0.1% formic acid/2% acetonitrile/98% H_2_O, loaded onto a C18 trap column (Thermo Scientific), and subsequently eluted from the trap column over the self-packed C18 analytic column in a 120 min gradient. The LC-MS/MS analysis was performed by using a Q Exactive HF instrument (Thermo Scientific) equipped with an Easy-nLC 1000 system. MS data was acquired and submitted to Proteome Discoverer 1.4 (Thermo Scientific) to perform protein identification and quantitation utilizing its integrated SEQUEST HT search engine and Percolator algorithm. The peptide mass tolerance was set to 10 ppm and 20 mmu for MS/MS. Carbamido methylation of cysteine was set as a fixed modification, and oxidation of methionine and deamidation of N, Q as a dynamic modification. A high confidence dataset with less than 1% FDR (false discovery rate) was used for peptide filtering. Files from the samples were searched against the *Arabidopsis* proteome database of Swiss-Prot (http://www.uniprot.org/).

### Proteinase K protection assay

The coding sequences (CDS), excluding the portion of the transit peptides, of *CPNA1*, *CPNA2*, *CPNB1*, *CPNB2*, *CPNB3*, *Cpn20*, and *KASI* in *Arabidopsis* were cloned into pET-28a (+) vector (Novagen). Then all the proteins were overexpressed in *E*. *coli* expression strain BL21 following induction with isopropyl β-D-thiogalactoside (IPTG), and the BL21 cells were harvested and resuspended in lysis buffer (50 mM Na_2_HPO_4_ pH 8.0, 0.3 M NaCl, 1% Triton X-100, 5% glycerol, 2 mM PMSF). Following sonication of the BL21 cells, the fusion proteins were purified by High Affinity Ni-NTA Resin (Genscript).

For the proteinase K protection assay, Cpn60s were reconstituted with Cpn60α and Cpn60β according to the method described previously [31,62]. 15 μM Cpn60α, 15 μM Cpn60β and 10 μM Cpn20 were mixed in the incubation buffer (50 mM Tris-HCl pH 8.0, 0.3 M NaCl, 10 mM MgCl_2_, 16 mM KCl, 2 mM DTT and 5 mM ATP), and then incubated for 2 h at 30°C. After centrifugation, the supernatant fraction of the reconstitution mixture was collected and loaded on a Enrich Size Exclusion Column 650 (Bio-Rad). Then the reconstituted Cpn60s were purified and collected by gel-filtration chromatography.

The proteinase K protection assay was performed according to the procedure as described previously [62] with some modification. The various purified Cpn60s (1 μM) and denatured substrate KASI (0.64 μM) were incubated in refolding buffer (50 mM Tris-HCl pH 7.5, 50 mM KCl, 5 mM MgCl_2_, 1.9 μM Cpn20 and 1 mM ADP) for 20 min at 25°C, and then proteinase K (sigma) was added to a final concentration of 2.0 μg/mL. After incubation for 0, 5, 10, 15 and 20 min at 25°C, the proteolysis was stopped by adding PMSF (2 mM). Finally, the content of the substrates in reaction mixtures were analyzed by SDS-PAGE and immunoblotting with His-tag antibody (Genscript).

### Immunoblotting analysis of KASI protein levels

Total protein in 7 and 14 DAG seedlings of WT and *cpna2-2* homozygous mutant carrying *ABI3pro*:*CPNA2-HA* vector was extracted according to the method described previously [59]. Then the concentrations of total protein were normalized by immunoblotting analysis of ACTIN using anti-ACTIN (ABclonal). 20 μl normalized protein samples were loaded on 12% SDS-PAGE gels and analyzed by immunoblotting using KASI antibody (ABclonal) to detect the KASI protein levels. The relative KASI protein levels in the seedlings of WT and *cpna2-2* homozygous mutant were quantified by ImageJ software.

### Phylogenetic analysis

Multiple sequence alignment of Cpn60α proteins in various species was generated with ClustalX 1.83 [63]. Then the alignment result was used for building the phylogenetic tree with MEGA 5.1 [64]. The neighbor-joining method was used with a bootstrap (1000 replicates) test of phylogeny.

### Homology modeling

The predicted structural models of CPNA1, CPNA2, BnaC02g08340D, LOC100257653 and L484_018489 were obtained by SWISS-MODEL (http://www.swissmodel.expasy.org/), while the crystal structure of GroEL (1AON, Chain A) was used as the template. The finished models were visualized using Swiss-Pdb Viewer 4.1.0 [65].

### Accession numbers

Sequence data in this article can be found in TAIR (The Arabidopsis Information Resource) under these accession numbers: *CPNA1* (AT2G28000), *CPNA2* (AT5G18820), *CPNB1* (AT1G55490), *CPNB2* (AT3G13470), *CPNB3* (AT5G56500), *CPNB4* (AT1G26230), *Cpn20* (AT5G20720), *KASI* (AT5G46290).

## Supporting information

S1 Fig Characterization of *cpna1* mutant in *Arabidopsis*. (A) Schematic diagram of *CPNA1* gene structure with the position of T-DNA insertion. (B) Phenotypic observation of embryos from wild-type plants and embryos in abnormal ovules from *cpna1*/+ plants. Bars = 20 μm.
(TIF)

S2 Fig Phenotypic observation of *cpnb* double mutants in *Arabidopsis*. (A) Schematic diagrams of *CPNB1*, *CPNB2*, *CPNB3* and *CPNB4* with the positions of T-DNA insertions. (B) Detection of transcript levels of *CPNB1-4* in *cpnb* homozygous mutants. Transcript levels were detected by reverse transcription PCR, and *GAPDH* was used as the control gene. (C) Silique phenotypes of *cpnb* double heterozygous mutants. Arrows indicate abnormal ovules. Abortion rates of siliques are shown on the right side. Bars = 1 mm.
(TIF)

S3 Fig Phylogenetic analysis of Cpn60α1 and Cpn60α2. The protein sequences used for phylogenetic analysis of Cpn60α1 and Cpn60α2 were obtained from NCBI with the Blastp program. The phylogenetic tree was constructed using the neighbor-joining method in MEGA 5.1. The red frames indicate Cpn60α proteins in *Arabidopsis*.
(TIF)

S4 Fig Sequence alignment of Cpn60α1 and Cpn60α2 in various species. CPNA2 and its orthologs are included in the green frames on the left side. The residues proposed to bind substrates by Fenton et al. [44] are below the red globules, and the residues proposed to bind substrates by Buckle et al. [42] are below the green globules. The amino acid residues in position 259 and 267 of Cpn60α1 and Cpn60α2 are included in the red frames. The black sequences have 100% homology, the red sequences have 75–100% homology, and the cyan sequences have 50–75% homology. The sequence alignment was conducted by DNAMAN 6.0.
(TIF)

S1 Table Segregation and genetic transmission of the *cpna2-2* allele in *Arabidopsis*. (DOCX)

S2 Table Functional partners of CPNA1 and CPNA2 predicted by *Arabidopsis thaliana* Protein Interactome Database. (DOCX)

S3 Table Distribution of embryo phenotypes in wild type and *CPNA2pro*:*amiR-KASI* transgenic lines at sequential development stages of *Arabidopsis* embryos. (DOCX)

S4 Table Primers (5’ to 3’) used in the experiments. (DOCX)

S1 Dataset MS analysis of the CPNA2, CPNA1 and WT immunoprecipitates. (XLSX)
