# Supplementary figures and images for: Functional divergence of chloroplast Cpn60α subunits during Arabidopsis embryo development
Source: PLoS Genet. 2017 Sep 29;13(9):e1007036. doi: 10.1371/journal.pgen.1007036 (PMC5636168; doi:10.1371/journal.pgen.1007036)

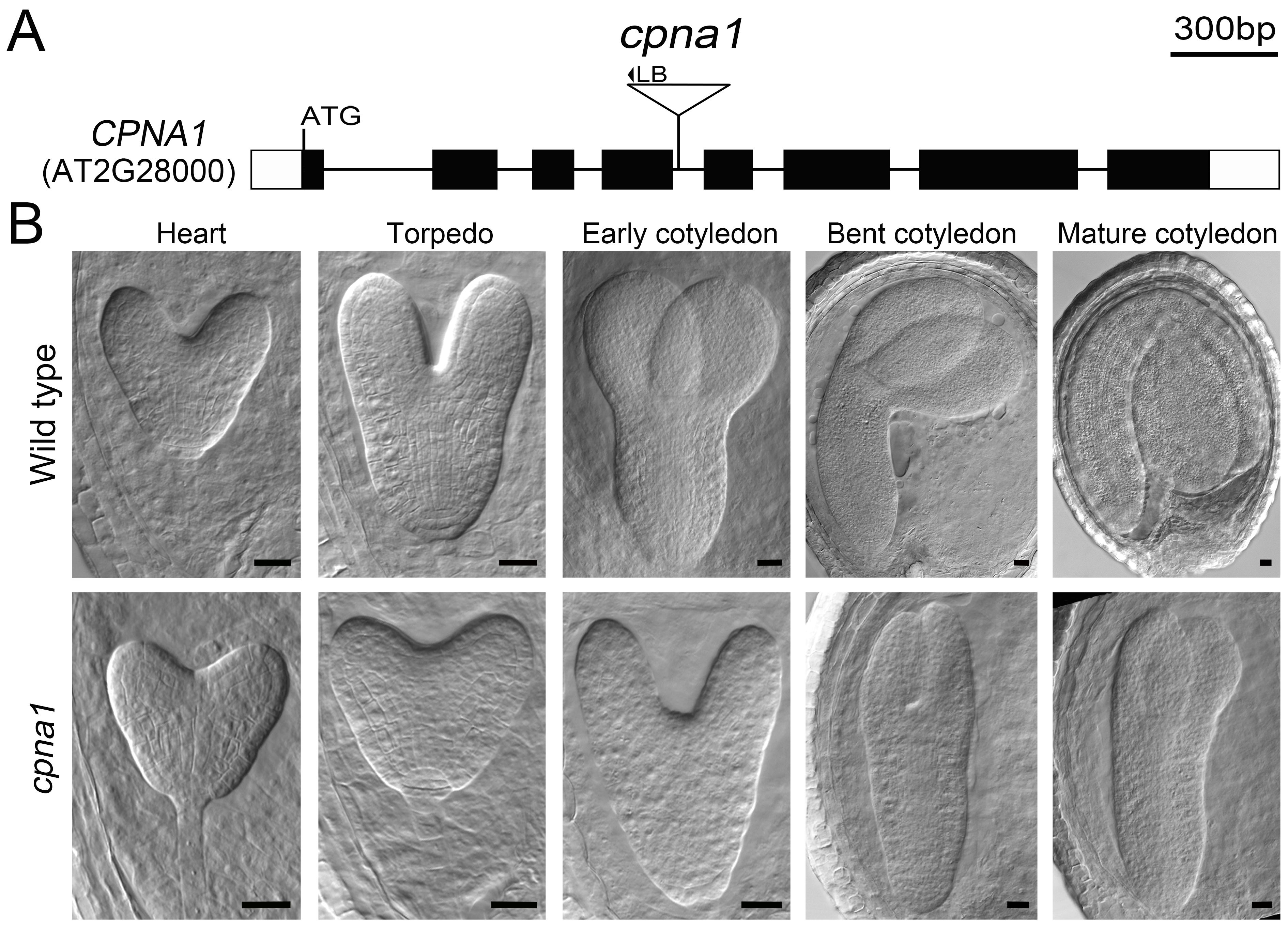

Supplement: S1 Fig — (A) Schematic diagram of CPNA1 gene structure with the position of T-DNA insertion. (B) Phenotypic observation of embryos from wild-type plants and embryos in abnormal ovules from cpna1/+ plants. Bars = 20 μm. (TIF) [file pgen.1007036.s001.tif]

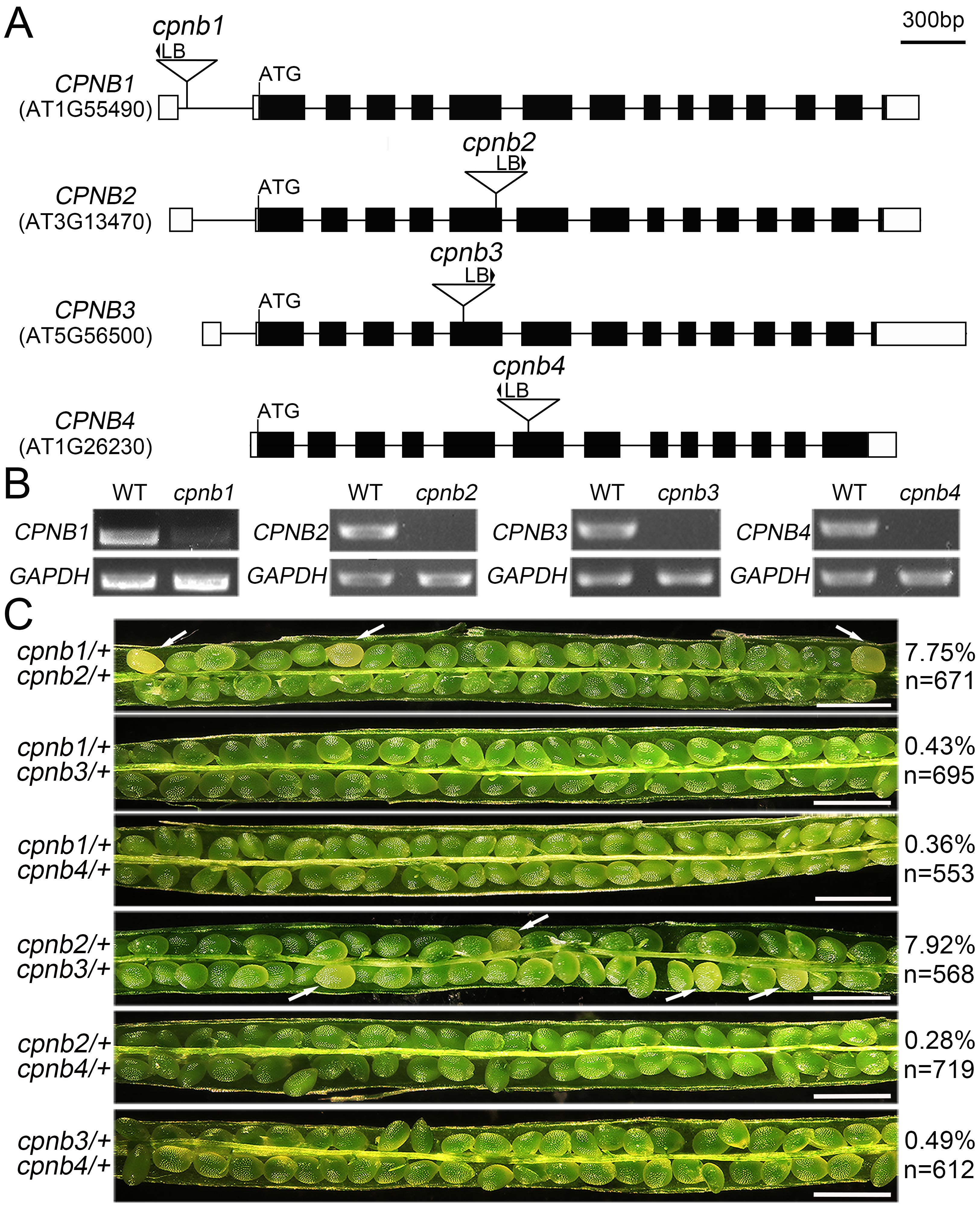

Supplement: S2 Fig — (A) Schematic diagrams of CPNB1, CPNB2, CPNB3 and CPNB4 with the positions of T-DNA insertions. (B) Detection of transcript levels of CPNB1-4 in cpnb homozygous mutants. Transcript levels were detected by reverse transcription PCR, and GAPDH was used as the control gene. (C) Silique phenotypes of cpnb double heterozygous mutants. Arrows indicate abnormal ovules. Abortion rates of siliques are shown on the right side. Bars = 1 mm. (TIF) [file pgen.1007036.s002.tif]

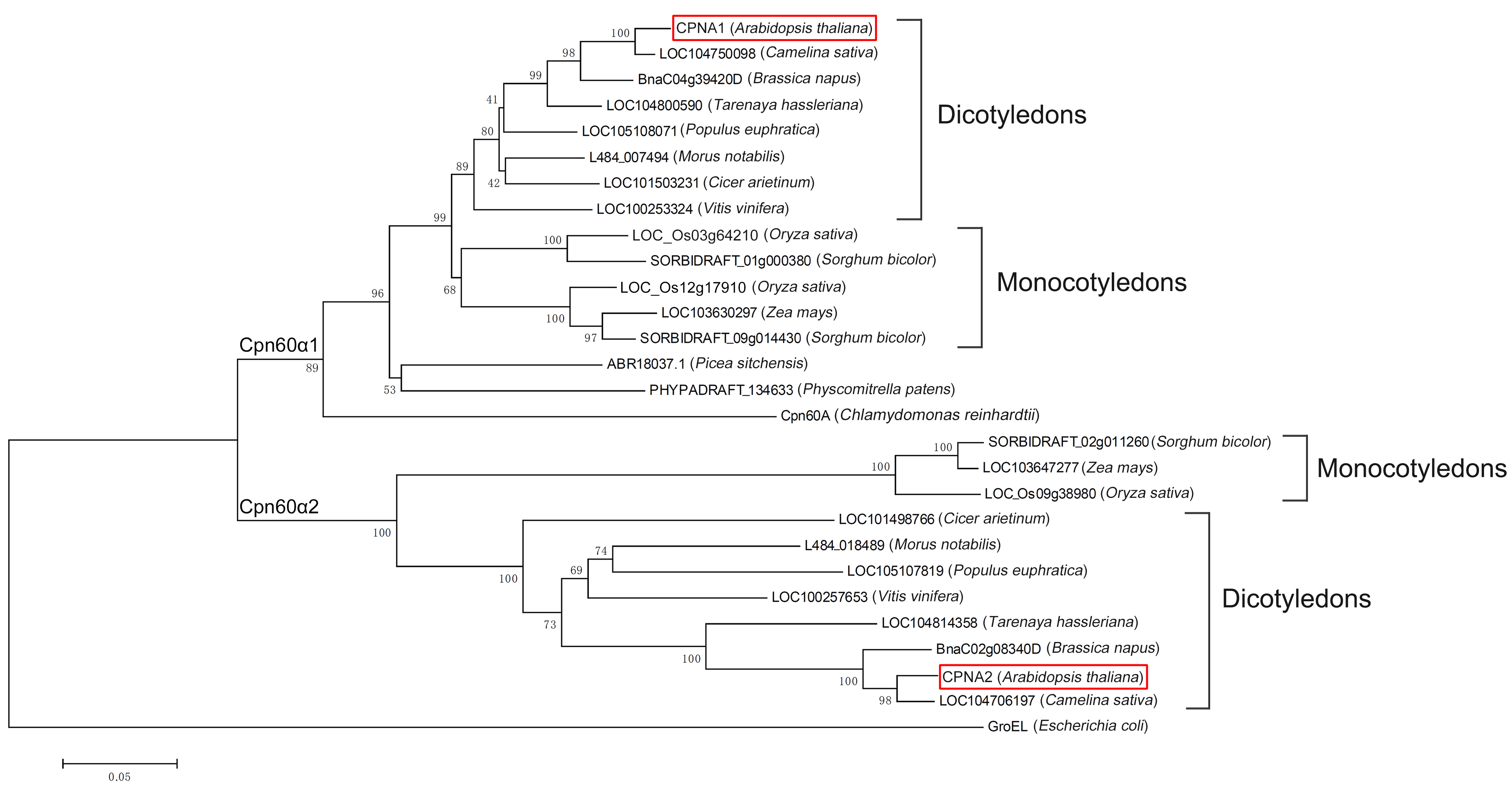

Supplement: S3 Fig — The protein sequences used for phylogenetic analysis of Cpn60α1 and Cpn60α2 were obtained from NCBI with the Blastp program. The phylogenetic tree was constructed using the neighbor-joining method in MEGA 5.1. The red frames indicate Cpn60α proteins in Arabidopsis. (TIF) [file pgen.1007036.s003.tif]

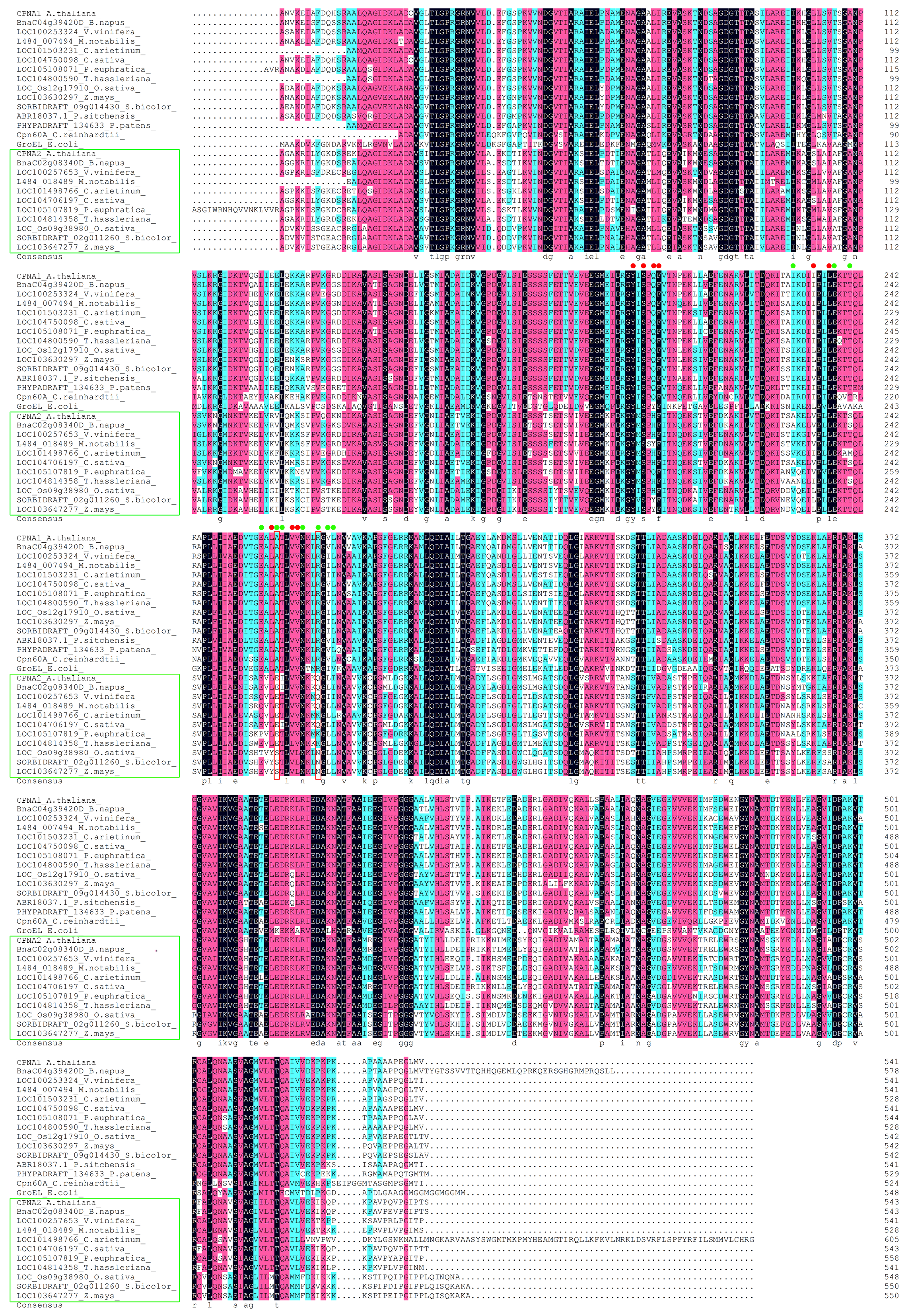

Supplement: S4 Fig — CPNA2 and its orthologs are included in the green frames on the left side. The residues proposed to bind substrates by Fenton et al. [44] are below the red globules, and the residues proposed to bind substrates by Buckle et al. [42] are below the green globules. The amino acid residues in position 259 and 267 of Cpn60α1 and Cpn60α2 are included in the red frames. The black sequences have 100% homology, the red sequences have 75–100% homology, and the cyan sequences have 50–75% homology. The sequence alignment was conducted by DNAMAN 6.0. (TIF) [file pgen.1007036.s004.tif]
