# Supplementary material for: Functional divergence of chloroplast Cpn60α subunits during Arabidopsis embryo development
Source: PLoS Genet. 2017 Sep 29;13(9):e1007036. doi: 10.1371/journal.pgen.1007036 (PMC5636168; doi:10.1371/journal.pgen.1007036)
Supplement: S1 Table — (DOCX) [file pgen.1007036.s005.docx]

**S1 Table. Segregation and genetic transmission of the *cpna2-2* allele in *Arabidopsis*.**

| Cross (Female×Male) | Progeny^a^ | | | Total | TE (%)^b^ | *P* value^c^ |
| --- | --- | --- | --- | --- | --- | --- |
|  | +/+ | *cpna2-2*/+ | *cpna2-2*/*cpna2-2* |  |  |  |
| *cpna2-2*/+ × *cpna2-2*/+ | 152 | 310 | 0 | 462 | NA^d^ | NA |
| *cpna2-2*/+ × +/+ | 141 | 147 | NA | 288 | 104 | 0.72 |
| +/+ × *cpna2-2*/+ | 120 | 114 | NA | 234 | 95 | 0.69 |

^a^Genotypes of progeny are determined by PCR analysis. ^b^TE (transmission efficiency) is calculated as the ratio of heterozygote to wild type. ^c^*P* value is calculated by Chi-square test for a 1:1 segregation hypothesis. ^d^Not applicable.
