# Supplementary material for: Functional divergence of chloroplast Cpn60α subunits during Arabidopsis embryo development
Source: PLoS Genet. 2017 Sep 29;13(9):e1007036. doi: 10.1371/journal.pgen.1007036 (PMC5636168; doi:10.1371/journal.pgen.1007036)
Supplement: S2 Table — (DOCX) [file pgen.1007036.s006.docx]

**S2 Table. Functional partners of CPNA1 and CPNA2 predicted by *Arabidopsis*** ***thaliana* Protein Interactome Database.**

| Cpn60α | Predicted functional partners | Gene locus | Total score^a^ |
| --- | --- | --- | --- |
| CPNA1 | AtCpn60β1 | AT1G55490 | 15512.23 |
|  | AtCpn60β2 | AT3G13470 | 15512.23 |
|  | AtCpn60β4 | AT1G26230 | 6187.06 |
|  | AtCpn60β3 | AT5G56500 | 1457.9 |
| CPNA2 | AtCpn60β3 | AT5G56500 | 39140.08 |
|  | AtCpn60β2 | AT3G13470 | 1533.26 |
|  | AtCpn60β1 | AT1G55490 | 1447.77 |
|  | AtCpn60β4 | AT1G26230 | 1246.24 |

^a^Total scores are calculated through ortholog interaction datasets, shared biological function, co-expression matrices, gene fusion method, gene neighbors method, phylogenetic profile method and enriched domain pair.
