# Supplementary material for: Functional divergence of chloroplast Cpn60α subunits during Arabidopsis embryo development
Source: PLoS Genet. 2017 Sep 29;13(9):e1007036. doi: 10.1371/journal.pgen.1007036 (PMC5636168; doi:10.1371/journal.pgen.1007036)
Supplement: S3 Table — (DOCX) [file pgen.1007036.s007.docx]

**S3 Table.** **Distribution of embryo phenotypes in wild type and *CPNA2pro:amiR-KASI* transgenic lines at sequential development stages of *Arabidopsis* embryos.**

| Parents | DAP | Total^a^ | The proportion (%) of embryos at different stages | | | |
| --- | --- | --- | --- | --- | --- | --- |
|  |  |  | G^b^ | TR/EH^c^ | LH/T^d^ | C^e^ |
| Wild type | 3 | 283 | 97.5 | 2.5 | ND^f^ | ND |
|  | 4 | 294 | 27.6 | 72.4 | ND | ND |
|  | 5 | 245 | ND | 22.4 | 77.6 | ND |
|  | 7 | 293 | ND | ND | 5.8 | 94.2 |
| amiR-16# | 3 | 329 | 96.7 | 3.3 | ND | ND |
|  | 4 | 257 | 25.7 | 74.3 | ND | ND |
|  | 5 | 241 | ND | 19.5 | 80.5 | ND |
|  | 7 | 311 | ND | ND | 3.9 | 96.1 |
| amiR-18# | 3 | 302 | 97.4 | 2.6 | ND | ND |
|  | 4 | 295 | 88.8 | 11.2 | ND | ND |
|  | 5 | 269 | 34.2 | 65.8 | ND | ND |
|  | 7 | 215 | ND | ND | 68.4 | 31.6 |
| amiR-23# | 3 | 308 | 97.7 | 2.3 | ND | ND |
|  | 4 | 269 | 90.3 | 9.7 | ND | ND |
|  | 5 | 230 | 29.6 | 70.4 | ND | ND |
|  | 7 | 268 | ND | ND | 63.8 | 36.2 |

^a^Number of the total counted embryos. ^b^Embryos at the globular stage. ^c^Embryos at the transition and early heart stages. ^d^Embryos at the late heart and torpedo stages. ^e^Embryos at the cotyledon stage. ^f^Not detected.
