# Supplementary material for: Functional divergence of chloroplast Cpn60α subunits during Arabidopsis embryo development
Source: PLoS Genet. 2017 Sep 29;13(9):e1007036. doi: 10.1371/journal.pgen.1007036 (PMC5636168; doi:10.1371/journal.pgen.1007036)
Supplement: S4 Table — (DOCX) [file pgen.1007036.s008.docx]

**S4 Table. Primers (5’ to 3’) used in the experiments.**

| Primers for genotypic analysis of T-DNA insertion mutants | | |
| --- | --- | --- |
|  | LP | RP |
| *cpna2-2* | gtggaccaaaccgttcaaac | atgttgctccagcgttctca |
| *cpna2-3* | tcaaacaaatccaatcttcgg | cttttcatctgcaacaccctg |
| *cpna1* | tctatggttgcgttttcaacc | ggaggaatgttgtgttggatg |
| *cpnb1* | tttgggtccaagtgtaacacc | aaaatttcgcaattaatcggg |
| *cpnb2* | catctcttcaagctcgtttgg | caacaagaagcaactgcacag |
| *cpnb3* | gagcaaatacggttcccctag | tttattgtcccacgaagcttg |
| *cpnb4* | agggcatcttcaacctttagc | tcatgttgcagcagttagtgc |
| LBb1.3 | attttgccgatttcggaac | |
| CSLB | gccttttcagaaatggataaatagc | |
| Primers for RT-PCR and qRT-PCR | | |
|  | FP | RP |
| CPNB1 | tgataagaaacttatcagcaag | tctcaagaacaacatttcgt |
| CPNB2 | agttgtcttctgcgactagca | tcttcaacctccttagacataagt |
| CPNB3 | gattgtcatcttttgtttcga | gacattttcttcaactcagcc |
| CPNB4 | tggaacaaatcccatccaag | cctaataacaggagcaagagcg |
| CPNA2 | ccgcgccgtagtggacag | agggcctaaggtgatgga |
| KASI | gagcacttcagggatcaaaatc | gccatccagtgttgatagcct |
| GAPDH | gagtctactggtgtcttcactg | caaggtcggacttgtattcgtg |
| Primers for *CPNA2pro:gCPNA2* | | |
|  | FP | RP |
| CPNA2-FL | cgcTCTAGAcatgagcgagttccaagaat | agaGGATCCctctctcgcttttttttttt |
| Primers for *CPNA2pro:GUS* | | |
|  | FP | RP |
| CPNA2pro | actGGATCCcatgagcgagttccaagaat | actAGATCTaccatttcgggtgtctcctctcct |
| Primers for *CPNA2pro:H2B-GFP* and *CPNA1pro:H2B-CFP* | | |
|  | FP | RP |
| CPNA2pro | cgcTCTAGAcatgagcgagttccaagaat | actGGATCCttcgggtgtctcctctcct |
| CPNA1pro | actCTGCAGgaataatgattgcgtaaagccg | actTCTAGAtttgagagaaaaggagggagc |
| CFP | actGGATCCatggtgagcaagggcgaggag | actGTCGACtcacttgtacagctcgtccatg |
| H2B | actGGATCCatggcgaaggcagataagaaac | actGGATCCagaactcgtaaacttcgtaacc |
| Primers for *35Spro:CPNA2-GFP* | | |
|  | FP | RP |
| CPNA2 | actGGATCCatgttcgccgtatcaccgt | actGGATCCtgatgtgggtatgccagga |
| Primers for Co-IP | | |
|  | FP | RP |
| CPNA2pro | cgcTCTAGAcatgagcgagttccaagaat | actGGATCCttcgggtgtctcctctcct |
| CPNA1pro | actCTGCAGgaataatgattgcgtaaagccg | actTCTAGAtttgagagaaaaggagggagc |
| CPNA2-HA | actGGTACCatgttcgccgtatcaccgt | actGTCGACctaagcgtaatctggaacatcgtatgggtatgatgtgggtatgccagga |
| CPNA1-HA | actGGTACCatggcgtctgcaaacgctct | actGTCGACctaagcgtaatctggaacatcgtatgggtacaccatgagaccctcaggag |
| Primers for prokaryotic expression | | |
|  | FP | RP |
| His-CPNA1 | actGCTAGCgctaatgtaaaggaaatagctt | actGAGCTCttacaccatgagaccctcagg |
| His-CPNA2 | actGCTAGCgctggagctaagagaatactat | actGAGCTCttatgatgtgggtatgccagg |
| His-CPNB1 | actGCTAGCtgcgcagcaaaggaattacat | actGAATTCttagtatccatatcctgagttgtc |
| His-CPNB2 | actGCTAGCtgtgcagccaaggagttgc | actGAATTCttagtatccataacctgagttgtc |
| His-CPNB3 | actGCTAGCtcaagctcttttggaaggact | actGAATTCttagaagccgtaacctgaattgt |
| His-Cpn20 | actGCTAGCgctgcttctgttgttgccc | actGAATTCctaagaaagtatagccatcacatc |
| His-KASI | actGCTAGCgcatccactgtctccgctc | actGAATTCtcagggtttgaaggcagaga |
| Primers for *ABI3pro:CPNA2-HA* | | |
|  | FP | RP |
| ABI3pro | actCTGCAGcggaaaatccgaggaaacca | actGGTACCcaaaacagtatcatcagcgt |
| ABI3FP | agatctagactcgaaaattt | |
| Primers for *CPNA2pro:amiR-KASI* | | |
| A | actggatccctgcaaggcgattaagttgggtaac | |
| B | actgagctccccatggcgatgccttaaataaa | |
| I | gatgatgtaatttacctccgcagtctctcttttgtattcc | |
| II | gactgcggaggtaaattacatcatcaaagagaatcaatga | |
| III | gactacggaggtaaaatacatcttcacaggtcgtgatatg | |
| IV | gaagatgtattttacctccgtagtctacatatatattcct | |
| Primers for site-directed mutation vectors of *CPNA2* | | |
| RP | cttcttgttcaccaccagtatctcaagcacttccgcagagata | |
| mRP | cttcttgttcaccaccagtatcgcaagcacttccgcagagata | |
| FP | gatactggtggtgaacaagaagcaaggtttgatcaatgtcgca | |
| mFP | gatactggtggtgaacaagaagagaggtttgatcaatgtcgca | |
